# Methylglyoxal-bis-guanylhydrazone inhibits osteopontin expression and differentiation in cultured human monocytes

**DOI:** 10.1371/journal.pone.0192680

**Published:** 2018-03-14

**Authors:** Xia Jin, Hua Xu, Michael S. McGrath

**Affiliations:** 1 Pathologica LLC, Burlingame, California, United States of America; 2 Department of Laboratory Medicine, Medicine, and Pathology, University of California San Francisco, San Francisco, CA, United States of America; INSERM, FRANCE

## Abstract

Monocyte activation and polarization play essential roles in many chronic inflammatory diseases. An imbalance of M1 and M2 macrophage activation (pro-inflammatory and alternatively activated, respectively) is believed to be a key aspect in the etiology of these diseases, thus a therapeutic approach that regulates macrophage activation could be of broad clinical relevance. Methylglyoxal-bis-guanylhydrazone (MGBG), a regulator of polyamine metabolism, has recently been shown to be concentrated in monocytes and macrophages, and interfere with HIV integration into the DNA of these cells *in vitro*. RNA expression analysis of monocytes from HIV+ and control donors with or without MGBG treatment revealed the only gene to be consistently down regulated by MGBG to be osteopontin (OPN). The elevated expression of this pro-inflammatory cytokine and monocyte chemoattractant is associated with various chronic inflammatory diseases. We demonstrate that MGBG is a potent inhibitor of secreted OPN (sOPN) in cultured monocytes with 50% inhibition achieved at 0.1 μM of the drug. Furthermore, inhibition of OPN RNA transcription in monocyte cultures occurs at similar concentrations of the drug. During differentiation of monocytes into macrophages *in vitro*, monocytes express cell surface CD16 and the cells undergo limited DNA synthesis as measured by uptake of BrdU. MGBG inhibited both activities at similar doses to those regulating OPN expression. In addition, monocyte treatment with MGBG inhibited differentiation into both M1 and M2 classes of macrophages at non-toxic doses. The inhibition of differentiation and anti-OPN effects of MGBG were specific for monocytes in that differentiated macrophages were nearly resistant to MGBG activities. Thus MGBG may have potential therapeutic utility in reducing or normalizing OPN levels and regulating monocyte activation in diseases that involve chronic inflammation.

## Introduction

Monocyte-derived macrophages can be classified as pro-inflammatory classical M1 or anti-inflammatory alternatively activated M2 macrophages in response to different stimuli [1, 2]. T-helper 1 (Th1) cytokine induced M1 macrophages release high levels of pro-inflammatory cytokines and are involved in microorganism and cell killing; while T-helper 2 (Th2) cytokine induced M2 macrophages are involved in phagocytosis of apoptotic cells, tissue remodeling, angiogenesis, and wound repair [2–4]. M1 and M2 macrophages are further classified into several subtypes with distinct differentiation markers [4]. The accumulated evidence indicates that an imbalance of macrophage polarization or activation is associated with chronic inflammatory diseases including rheumatoid arthritis, atherosclerosis, cancer, obesity, diabetes, and various neurodegenerative conditions such as macular degeneration and HIV-associated dementia (HAD) [5–7]. Controlling monocyte activation and trafficking might provide a significant therapeutic route for treatment of these chronic diseases.

Monocytes are an essential component of the innate immune system and are critically involved in the initiation of an adaptive immune response. Human peripheral blood monocytes can be broadly classified into two groups dependent on their level of expression of the low-affinity Fc receptor CD16 [8–11]. The majority of human monocytes (~ 80%) express little or no CD16 and high levels of the chemokine receptor 2 (CCR2). These “classical” monocytes migrate in response to monocyte chemoattractant protein 1 (MCP1 also designated CCL2) and can be induced to differentiate into tissue macrophages or dendritic cells *in vitro* if provided a suitable surface for attachment in concert with exposure to the macrophage colony stimulating factor (M-CSF) or other factors [11–13]. The remaining 10–20% of human blood monocytes express high levels of CD16 and little or no CCR2. These cells have been demonstrated to migrate in response to fractalkine (CXCL3) [14]. Early studies have shown that CD16 expression is up-regulated by culturing human monocytes, and that CD14+CD16+ monocytes may be derived from the CD14+/CD16- fraction [15, 16]. CD14+CD16+ monocytes exhibit features of tissue macrophages, and have been labeled as pro-inflammatory based on their high level production of the pro-inflammatory cytokine tumor necrosis factor (TNF) and low levels of the anti-inflammatory cytokine interleukin 10 (IL10) [9, 17–19]. Increased numbers of blood CD14+CD16+ monocytes are observed in a number of viral and autoimmune disorders including rheumatoid arthritis, HAD, amyotrophic lateral sclerosis (ALS), Alzheimer’s disease, and some malignancies [20–25]. Furthermore, there is increasing evidence suggesting that CD14+CD16+ monocytes may serve as HIV reservoirs in AIDS patients, promoting ongoing HIV infection [23, 26, 27]. For all these reasons, the *in vitro* and *in vivo* properties and function of these CD14+CD16+ monocytes are of considerable interest.

In studies on the relationship of macrophage activation to chronic disease, attention has been drawn to OPN as a critical cofactor. OPN is an extracellular matrix glycoprotein that influences multiple physiological functions *in vivo*, including bone metabolism, immune regulation, wound healing, cell survival and tumor progression [28–31]. Accumulating evidence indicates that OPN is a pro-inflammatory cytokine broadly involved in chronic inflammatory diseases, obesity, diabetes, autoimmune diseases, and neurodegenerative disorders [32, 33]. OPN is expressed in many cell types and tissues including activated macrophages and T cells [32]. Various OPN isoforms have been identified, and are apparently the result of alternative splicing, alternative translation, and post translational modifications [34]. In addition to secreted forms of OPN, intracellular OPN (iOPN) is found in the cytoplasm and the nucleus and has functions distinct from secreted OPN (sOPN) [35]. High levels of OPN expression in tissue and blood have been reported in patients with cancer [36, 37], infectious diseases such as HAD [38, 39], autoimmune disorders including multiple sclerosis and rheumatoid arthritis [40–42], obesity and diabetes [43, 44]. Studies of elevated OPN levels in inflammatory wounds reveal that OPN functions as an inflammation mediator and macrophage functional regulator [30, 31]. OPN exhibits Th1 cytokine functions in cell-mediated immunity assays [45]. Therapeutic approaches utilizing OPN small interfering RNA and anti-OPN neutralizing antibodies have been employed in cancer and inflammatory diseases [46–50] and have demonstrated the potential importance of OPN regulation as a therapeutic target.

Polyamines are required for cell differentiation and proliferation in general [51, 52]. Inhibitors of polyamine biosynthesis interfere with TNF-induced macrophage activation [53, 54], and therefore are thought to have potential value in controlling pathological inflammation associated with activated macrophages. MGBG, a polyamine biosynthesis inhibitor, is known to interfere with polyamine biosynthesis via the inhibition of the enzyme s-adenosylmethionine decarboxylase (SAMDC) [55–57]. Our recent studies have shown that MGBG is taken up specifically by monocytes and macrophages, and it inhibits HIV DNA integration and HIV expression in macrophages [58]. Gene expression studies performed on both HIV+ and healthy cultured peripheral blood mononuclear cells (PBMCs) showed that one gene, OPN, was consistently down regulated after overnight exposure to MGBG [59]. In this study, we show that MGBG regulates expression of OPN at both RNA and secreted protein levels in monocytes and also inhibits monocyte differentiation into M1 and M2 macrophage subsets without apparent effect on mature macrophage OPN expression.

## Materials and methods

### Cell culture and macrophage polarization

Human buffy coats or heparinized blood were obtained from blood donors from the Stanford Blood Center. PBMCs were isolated by density gradient centrifugation using Percoll or Ficoll-Paque Plus (GE Healthcare Life Sciences, Pittsburgh, PA). Monocytes were isolated from PBMCs using anti-CD14 (Miltenyi Biotech, Auburn, CA) MACS magnetic separation system. PBMCs or sorted monocytes were cultured in RPMI 1640 containing L-glutamine supplemented with 10% fetal bovine serum (FBS) and 1 mM sodium pyruvate. MGBG (Ash Stevens, Riverview, MI) and spermine (SPM) (Sigma-Aldrich, St. Louis, MO) were added where specified. FBS was replaced by 5% human serum when SPM was used. 10 ng/ml M-CSF (R&D Systems, Minneapolis, MN) was supplemented to aid the maturity of macrophages in culture. Cells were incubated at 1 million cells per ml medium unless otherwise indicated under suspension culture in 50 ml conical polypropylene tubes or 5 ml BD Falcon polypropylene tubes at 37°C in a humidified 5% CO2 incubator. After incubating for a designated number of days, cells were collected for flow cytometry and total RNA preparation, and culture supernatants were saved for cytokine measurement using enzyme-linked immunosorbent assay (ELISA). To induce monocytes into M1 or M2 macrophages, isolated monocytes were incubated with 50 ng/ml interferon γ (INF-γ) and 1 μg/ml liposaccharides (LPS) or 50 ng/ml interleukin 4 (IL4) (Sigma-Aldrich, St. Louis, MO), respectively, for 5 to 6 days for differentiation and polarization.

### Quantitative real-time PCR analysis

Cultured monocytes were spun down and lysed in Trizol reagents (Thermo Scientific, Waltham, MA), and total RNA prepared using the PureLink Micro to Midi total RNA purification systems (Thermo Scientific, Waltham, MA). 100 ng of total RNA was converted to cDNA using the Verso cDNA Synthesis kit (Thermo Scientific, Waltham, MA) by incubating at 42°C for 1 hour followed by 95°C for 2 minutes. PCR was performed on the Agilent Mx3000P qPCR system using ABsolute Blue qPCR SYBR Kit (Thermo Scientific, Waltham, MA) and 200 ng cDNA in a 25 μl reaction. Thermal amplification profile included one cycle of template denaturation at 95°C for 15 minutes followed by 40 cycles of 95°C for 15 seconds, 60°C for 30 seconds, and 72°C for 30 seconds. The presence of a single amplified product was confirmed by DNA melting point analysis. Threshold cycles (Ct) for each amplification reaction were determined using respective software for the instrument. All samples were amplified with the human β-actin LightCycler—Primer Set (Roche Diagnostics, Indianapolis, IN) and the OPN primers (Forward: 5’- AGC CAC AAG CAG TCC AGA TTA T and Reverse: 5’- TTG ACC TCA GAA GAT GCA CTA TC). Results with the OPN primers for individual samples were normalized to signals obtained with β-actin from the same sample (Ct _actin_—Ct _OPN_). Gene expression fold change was calculated (2^Δ(Ct actin–Ct OPN)^).

### Flow cytometry analysis

- Macrophage differentiation study. Monocytes or macrophages were double stained for differentiation marker expression. One million freshly isolated or cultured cells were incubated with 10 μl of peridinin chlorophyll protein (PerCP) conjugated anti-CD14 monoclonal antibody (BD Biosciences, San Jose, CA) and fluorescein isothiocyanate (FITC) or phycoerythrin (PE) conjugated monoclonal antibodies against macrophage differentiation markers for 20 minutes at room temperature in 100 μl phosphate buffered saline (PBS). Matching isotype antibody staining was performed in parallel for cell population gating. After washing and fixing, fluorescent emission was measured using a BD FACScan flow cytometer (Becton Dickinson, Franklin Lakes, NJ). 10,000 to 50,000 cells were analyzed per sample. Cell debris and main subpopulations of the cells were gated using forward and side scatter (FSC and SSC, respectively) plots. Cells were further gated according to isotype antibody staining (S1 Fig). Geometric mean fluorescence or percentage of the cell population was obtained for the gated cells.
- Intracellular staining of iONP. Macrophage iOPN expression was measured using the Inside Stain Kit (Miltenyi Biotec, San Diego, CA). Cultured cells were stained with surface antibodies PerCP-CD14 and FITC-CD16 followed by iOPN staining using the Inside Stain kit and PE conjugated anti-OPN antibody (BD Biosciences, San Jose, CA) according to manufacturer’s instructions.
- BrdU incorporation study. Monocyte BrdU uptake or DNA synthesis was determined using a FITC BrdU flow kit (BD Biosciences, San Jose, CA). Sorted monocytes were cultured and pulsed with 10 μM BrdU for 3–6 days supplemented with 100 ng/ml M-CSF. Cells were also treated with or without MGBG. After culture, the cells were collected and stained with PerCP conjugated anti-CD14 antibody. The percentage of BrdU positive cells were measured after BrdU staining using the FITC BrdU flow kit according to manufacturer’s instructions.

### Cytokine assay

Cell culture supernatants were collected for sOPN cytokine measurement. Cytokine levels were measured using a Human OPN Quantikine ELISA kit (R&D Systems, Minneapolis, MN) according to manufacturer’s instructions. Each sample was tested in duplicates.

### Statistical analysis

A two-tailed paired t-test was used to determine statistical differences, and P < 0.05 was considered significant (S1 Table). All the experiments were carried out for at least three times unless otherwise indicated. Data were presented as means and SEM.

## Results

### MGBG inhibits both OPN RNA expression and secreted protein levels in a dose-dependent manner in normal monocyte cultures

Previous gene expression screening studies in PBMC preparations from HIV infected and healthy individuals showed that of the genes affected by MGBG, OPN was the only gene consistently down regulated in all specimens [59]. Considering that monocytes and macrophages are the only blood cells capable of taking up significant amounts of MGBG [58] and the importance of OPN as a macrophage produced factor implicated in the pathogenesis of HIV disease and the chemotaxis of monocytes into diseased tissues [60–62], most subsequent studies were carried out in these cell populations and focused on the role that MGBG might play in regulation of OPN expression.

**Fig 1:**
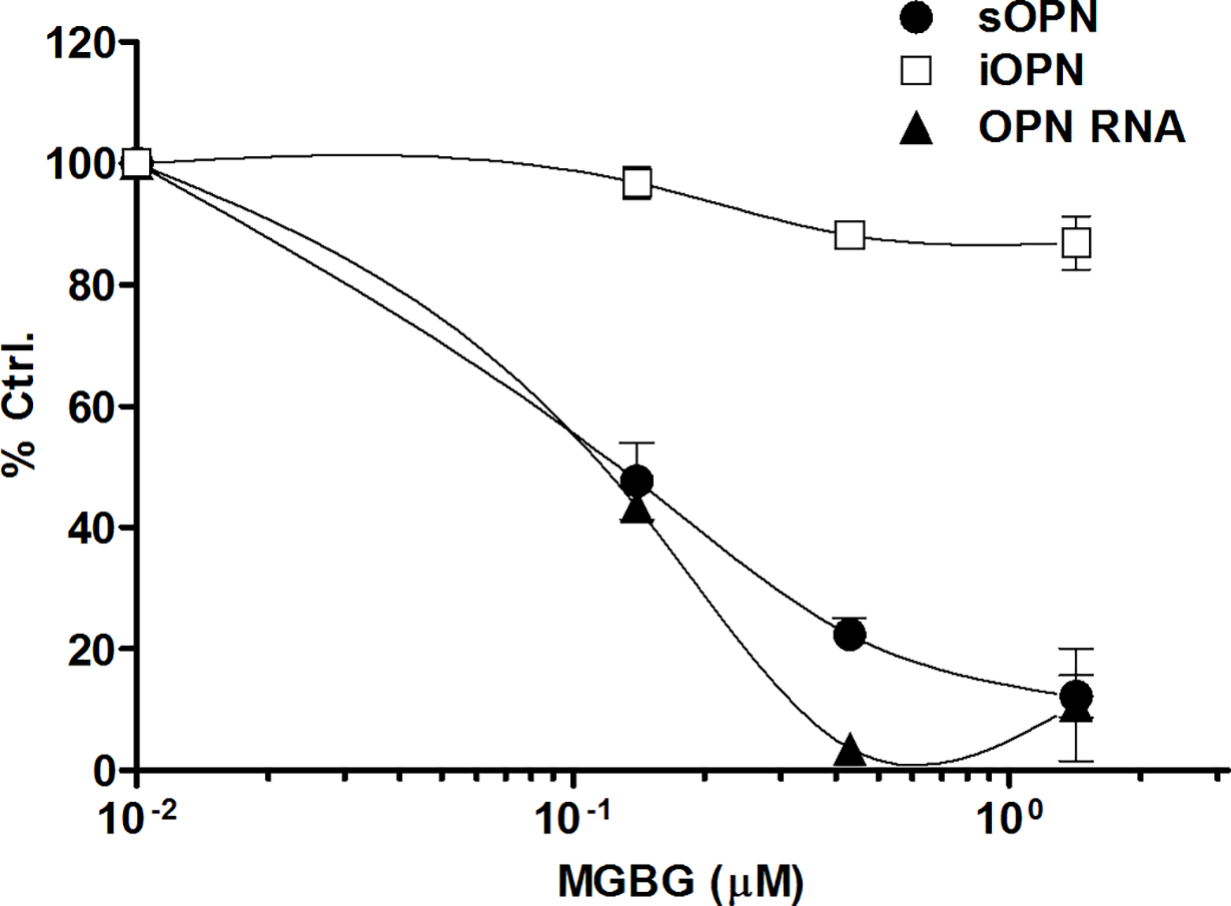
MGBG inhibited sOPN levels and gene expression in cultured human monocytes. 1 day cultured monocytes were collected for total RNA extraction and iOPN expression, and culture supernatants for OPN ELISA; quantitative real-time PCR was performed for OPN gene expression. iOPN expression was measured by flow cytometry. MGBG decreased OPN gene expression and sOPN level in a dose-dependent manner. MGBG slightly decreased iOPN expression. The data were normalized against that of untreated controls. The average sOPN level for 1 day untreated monocytes was approximately 3 ng/ml. The iOPN geometric mean florescence of 1 day untreated cells varies among individuals ranging from 209–689. The OPN RNA level was calculated from β-actin normalized Ct difference of 1D cultured cells versus cells at isolation. The OPN Ct difference of 1 day untreated monocytes also varies among individuals ranging from 1.7 to 9.9. ED50 of MGBG on OPN cytokine level and gene expression on day 1: ~ 0.1 μM, n = 4, means and SEM.

**Fig 2:**
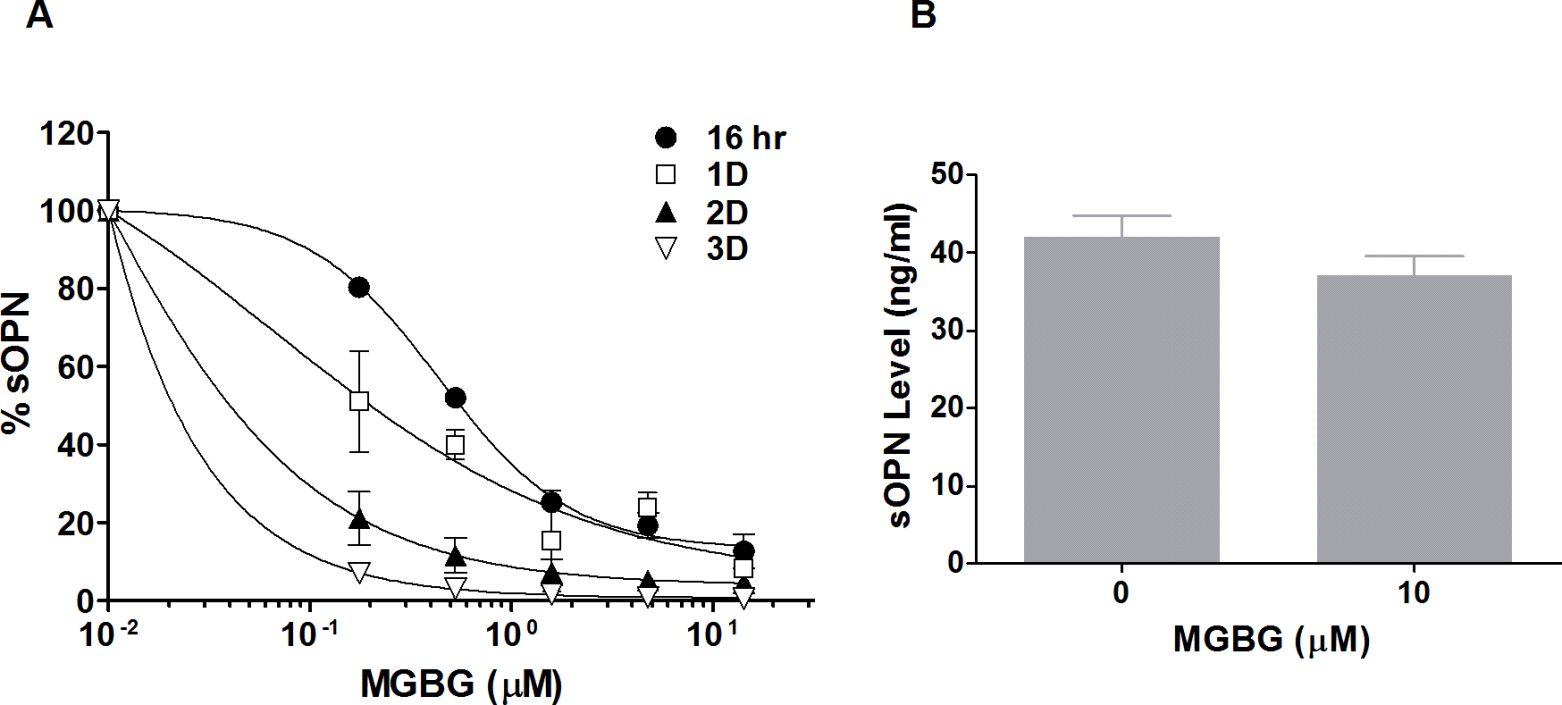
MGBG potently inhibited sOPN production in monocytes, but showed limited inhibitory effect in differentiated macrophages. (A). MGBG significantly inhibited sOPN production in monocytes. The degree of MGBG inhibition on sOPN was related to time of exposure. Isolated human monocytes were cultured at 0.1 million cells per ml with various concentrations of MGBG for 16 hr, 1, 2 and 3 days. MGBG showed potent inhibition on monocyte sOPN production with IC50 < 0.1 μM on day 3. Data were presented as percentage of untreated controls. The average sOPN levels of untreated monocytes were approximately 110, 130, 670 and 5300 pg/ml at 16 hr, 1, 2, and 3 days, respectively. The inhibition is independent of cytotoxicity (e.g. little cell death at 16 hours and day 1). The data is normalized to number of live cells in the culture. n = 2. (B). MGBG showed limited inhibitory effect on sOPN production in mature macrophages. Isolated monocytes were cultured to allowed to differentiate into macrophages for 6 days prior to drug treatment. Fresh media was replaced and cells were treated with 10 μM MGBG for 3 days. n = 4, means and SEM.

To further investigate the effects of MGBG on the production of OPN, various doses of the drug were incubated in cultures of purified monocytes isolated from healthy individuals. Fig 1 shows that OPN RNA and secreted protein (sOPN) but not iOPN levels were significantly reduced (p < 0.005) by MGBG treatment in a dose-dependent manner in 1 day cultured monocytes. As in earlier studies of HIV infection, the regulation of OPN expression occurred at submicromolar levels of MGBG, and there were no observed toxicities at the drug doses tested. The 50% effective dose (ED50) of MGBG on OPN RNA expression and OPN protein level after one day of treatment was observed at approximately 0.1 μM. The effect of MGBG on OPN expression was also related to time of exposure with longer exposures associated with more profound inhibition of sOPN expression as shown in Fig 2A. In the performance of monocyte cultures with addition of MGBG at different time points after culture initiation, it was noted that the OPN regulatory effect was reduced when MGBG was added to monocyte cultures at later time points. In contrast to the regulation of sOPN by MGBG in monocyte cultures, mature macrophage (after six days of monocyte cultivation) production of sOPN was not significantly affected even at high concentrations of MGBG (Fig 2B).

### MGBG regulates activation of cultured monocytes

**Fig 3:**
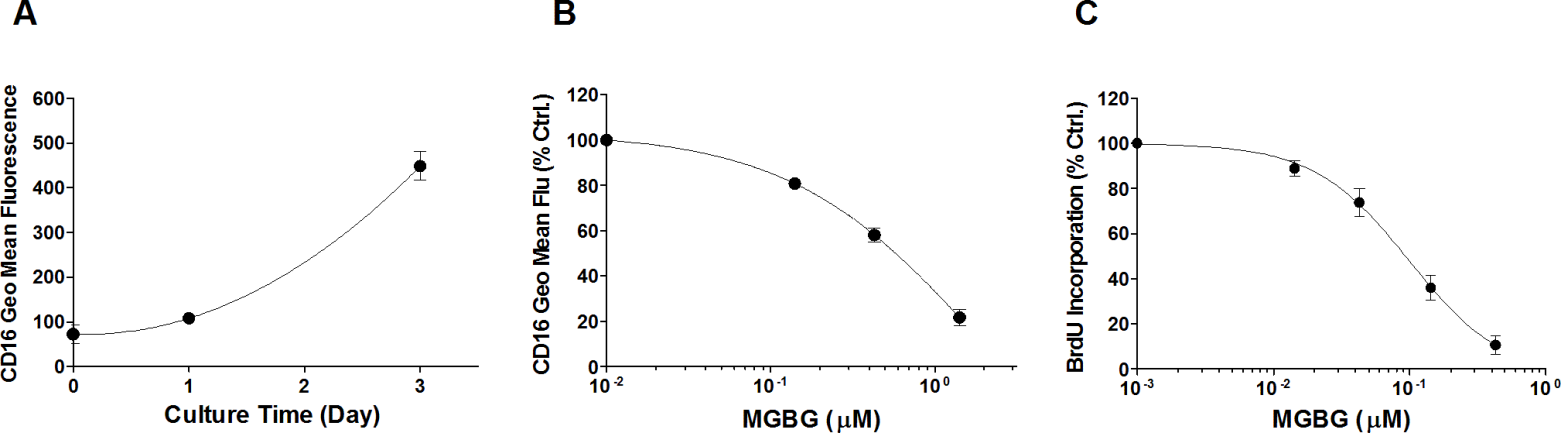
MGBG inhibited monocyte CD16 expression and BrdU incorporation. Isolated human monocytes were cultured in suspension with or without MGBG treatment. CD16 expression was measured by flow cytometry. (A). Monocytes differentiate spontaneously in culture. CD16 expression level increases in monocyte culture. n = 4, means and SEM. (B). MGBG inhibited CD16 expression in 1 day cultured monocytes in a dose-dependent manner. The average CD16 geometric mean fluorescence of 1 day untreated monocytes was measured at 108 units. n = 4, means and SEM. (C). MGBG inhibited BrdU incorporation in cultured monocytes. Isolated human monocytes were cultured for 3–6 days for BrdU incorporation using a FITC BrdU flow kit. The average percentage of BrdU incorporated untreated monocytes was measured at 4.7%. MGBG inhibited monocyte BrdU uptake in a dose-dependent manner. n = 5, means and SEM.

**Fig 4:**
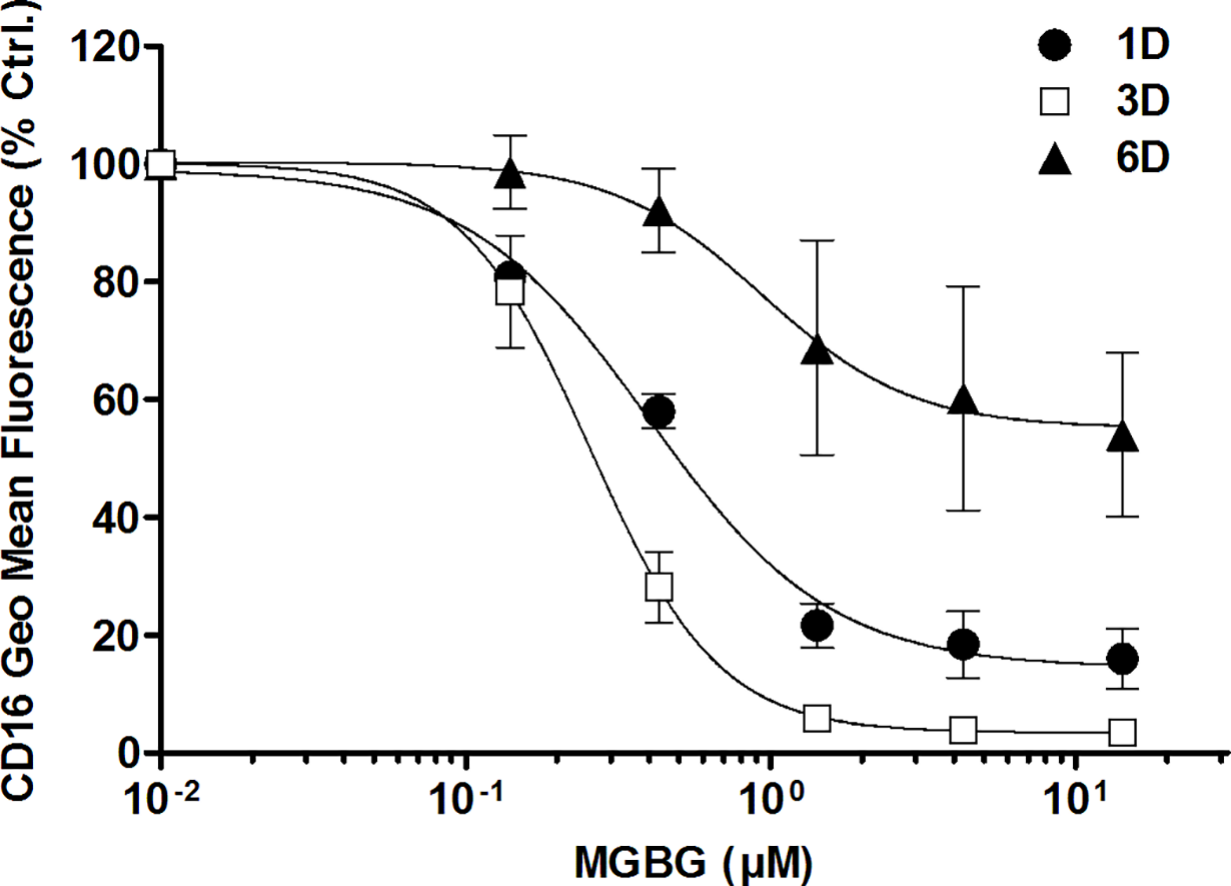
MGBG showed greater inhibition on CD16 expression in monocytes than that in differentiated macrophages. Monocytes were isolated from PBMCs using CD14 microbeads. Cells were cultured for 1, 3, and 6 days with various concentrations of MGBG, and collected for CD16 expression measurement using flow cytometry. MGBG showed greater inhibitory effect on CD 16 expression in 1 and 3 day monocytes than that in differentiated macrophages. The average CD16 geometric mean fluorescence was measured at 108, 448, and 249 for 1, 3, and 6 day untreated cells, respectively. n = 4, means and SEM.

**Fig 5:**
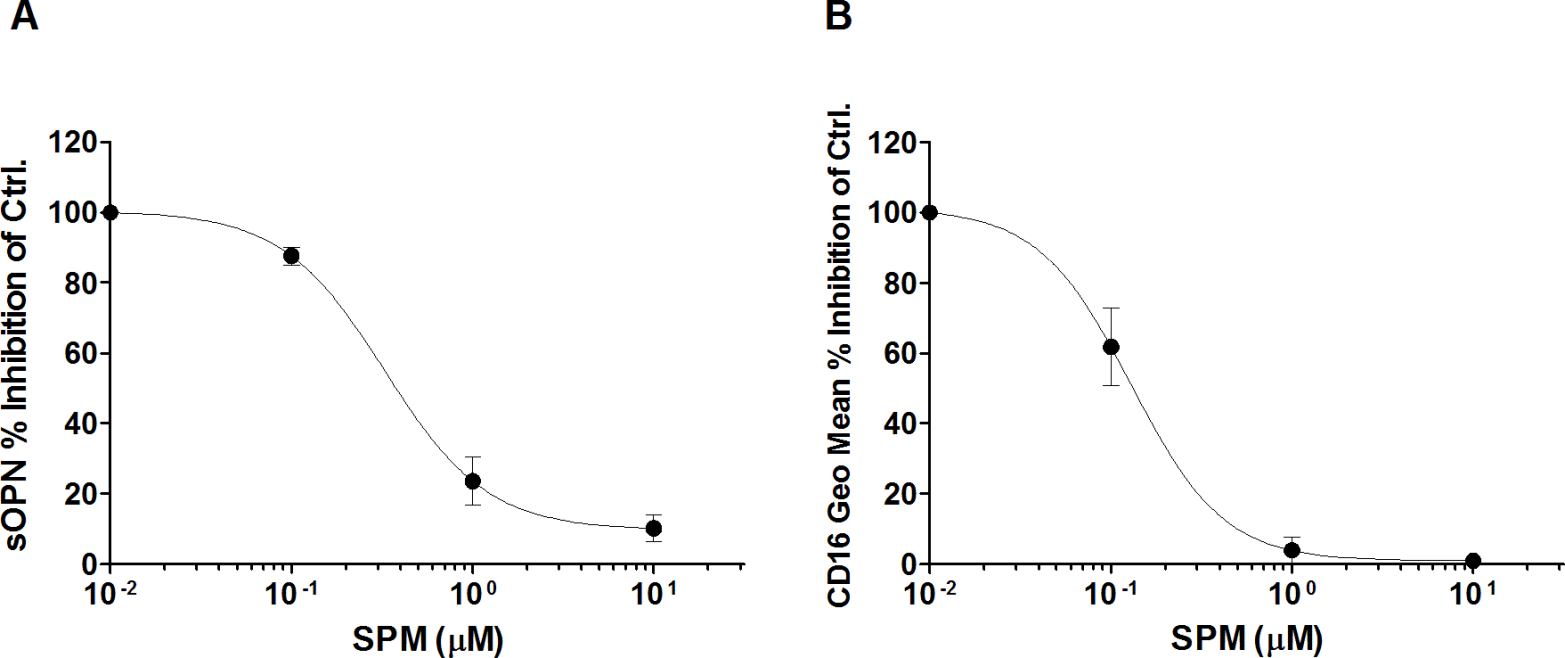
SPM reversed the MGBG inhibitory effects on sOPN and CD16 expression in monocytes. PBMCs were cultured for 3 days with or without treatment. Cells were cultured with 10 μM MGBG and various concentrations of SPM. (A). MGBG significantly decreased sOPN; SPM reversed the MGBG inhibitory effects on sOPN. The average sOPN level of 3 day untreated and 10 μM MGBG treated cells was 30 and 2 ng/ml, respectively. n = 6, means and SEM. (B). MGBG inhibited monocyte CD16 expression; SPM reversed the MGBG inhibitory effects on CD16 expression. Cultured PBMCs were double stained with CD14 and CD16 antibodies for flow cytometry analysis. The average CD16 geometric mean in untreated and MGBG treated CD14+ monocytes were measured at 510 and 249, respectively. n = 3, means and SEM. Data was presented as a percentage of inhibition of MGMG treatment only.

Monocyte activation *in vitro* is associated with up-regulation of CD16, a differentiation marker that is expressed on monocytes trafficking to tissue-based sites of inflammation [9, 17–19]. *In vitro* activation is also associated with short term uptake of BrdU by monocytes in the course of this differentiation process [63]. A small fraction (4–5%) of cultured monocytes was observed taking up BrdU during this process. Fig 3A shows that CD16 expression spontaneously increased in cultured monocytes whereas this aspect of cell differentiation was inhibited in a dose-dependent manner in cultures exposed to MGBG (Fig 3B). In parallel experiments, BrdU uptake in monocytes was similarly inhibited by MGBG in 3–6 day monocyte cultures (Fig 3C). In contrast, OPN expression in macrophages was relatively unaffected by MGBG (Fig 2B). When monocyte cultures were allowed to differentiate into macrophages *in vitro*, a time dependent decrease in regulation of CD16 expression by MGBG was observed: 6 day old macrophages were relatively unaffected by MGBG (Fig 4). Both the regulation of OPN and CD16 expression by MGBG were progressively reversed by exogenous addition of increasing amount of SPM, consistent with the regulation being mediated by MGBG uptake through the polyamine transporter (Fig 5).

### MGBG treatment interferes with induction of monocytes into polarized macrophages

**Fig 6:**
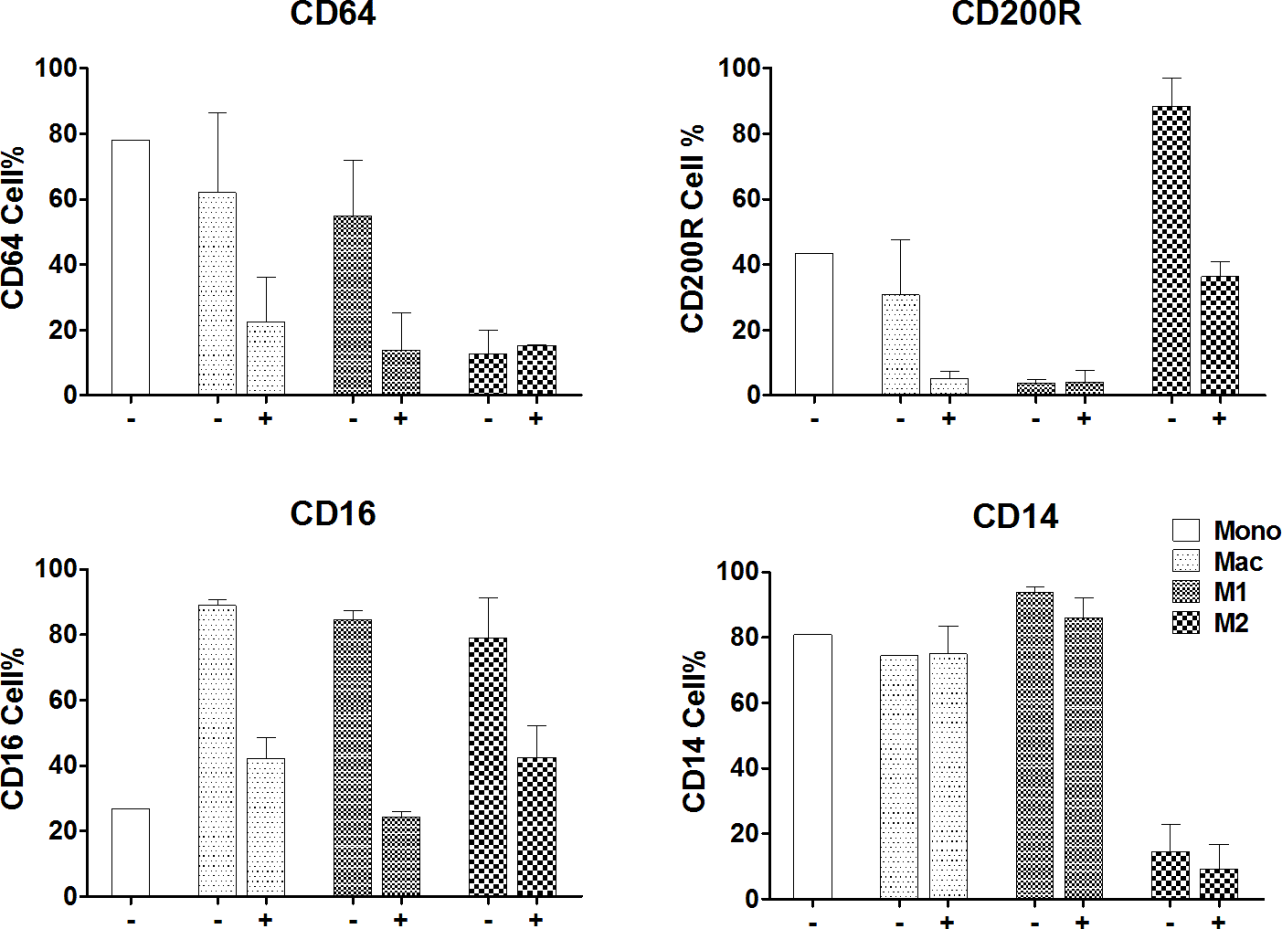
MGBG inhibited polarization of monocytes into M1 and M2 macrophages. Isolated human monocytes were cultured to differentiate and induced to M1 or M2 macrophages using 50 ng/ml INF-γ and 1 μg/ml LPS or 50 ng/ml IL4 for 5–6 days with or without 0.4 μM MGBG. Cells were cultured and evaluated by flow cytometry at day 5 or 6. Mono: Baseline expression of cell surface markers in 4 hr cultured monocytes (% of all monocytes); Mac: Cultured in normal media -/+ 0.4 μM MGBG; M1: Cultured in M1 polarizing media -/+ 0.4 μM MGBG; M2: Cultured in M2 polarizing media -/+ 0.4 μM MGBG. MGBG inhibited monocyte differentiation in both M1 and M2 macrophages. n = 2.

Monocyte activation leads to two functional forms of macrophages: those involved in inflammation termed M1 and those more associated with suppressive or M2 functions. To test whether MGBG would preferentially regulate or affect monocyte activation/differentiation into one of these macrophage subsets, monocytes were cultured in media that promoted either M1 or M2 macrophage differentiation with or without 0.4 μM MGBG treatment for 5–6 days. The culture conditions induced characteristic changes consistent with macrophage polarization. INF-γ and LPS induced M1 macrophages appeared elongated whereas IL4 induced M2 macrophages appeared more rounded compared to non-induced controls. Fig 6 shows the effects of MGBG on macrophage differentiation markers in polarized macrophages. In each panel, expression of monocyte antigens or baseline differentiation markers, non-polarized control macrophages, M1 or M2 polarized macrophages with or without drug treatment are presented as a percentage of the untreated population. The results show that MGBG treatment inhibited differentiation of monocytes into both M1 and M2 macrophage phenotypes. MGBG inhibited monocyte expression of CD64, a characteristic marker of M1 macrophages. In parallel cultures for M2 induction, MGBG interfered with the initiation of expression of M2 marker CD200R as well. In contrast to the effects on differentiation exhibited by MGBG, there was no significant effect on regulating expression of the pan monocyte/macrophage marker CD14 in parallel cultures.

## Discussion

Macrophage activation plays a pivotal role in chronic inflammation which is fundamental in the etiology of many diseases. Regulating the activation of macrophages could lead to a means of prevention or treatment of these diseases. MGBG, a compound that inhibits polyamine biosynthesis, inhibits TNF-induced macrophage activation [54], HIV proviral DNA integration in macrophages [58], and OPN gene expression in PBMCs [59]. In this study, we further investigated the drug’s effects on monocyte differentiation and OPN production.

Previous studies have shown that MGBG is selectively taken up by monocytes and macrophages [58]. These cells are also the main source of OPN in non-stimulated PBMC cultures (S11 Fig). Thus, the effects of MGBG on OPN expression in PBMC preparations most likely represent the effects of the drug on monocytes and macrophages. Studies carried out in purified monocytes further confirmed that MGBG’s inhibitory effect on OPN occurs at the RNA transcription level (Fig 1). This inhibitory effect was accompanied by the loss of secretory OPN in culture supernatants. This effect was restricted to sOPN since iOPN levels were largely unaffected in these short term assays. Furthermore, MGBG’s inhibitory effect on sOPN production was more pronounced in longer term of exposures (Fig 2A). This inhibitory effect was reversed by the addition of SPM. In Fig 5A, 1 μM SPM was effective in attenuating the inhibitory effect of 10 μM MGBG on sOPN production. SPM treatment alone showed no significant effects on monocyte OPN expression. MGBG is taken up by monocytes through the polyamine transporter in a temperature dependent manner, and SPM acts as a competitor of MGBG uptake [58]. This experiment confirms our conclusion that the MGBG activity is dependent on the polyamine transporter, as previously described for the anti-HIV activities of MGBG.

Freshly isolated blood CD14+CD16- monocytes differentiate into CD14+CD16+ monocytes during *in vitro* culture [15, 16] Fig 2A). Pro-inflammatory CD14+CD16+ monocytes play an essential role in infection and inflammation [20–25]. In this study, MGBG treatment of cultured monocytes resulted in a dose-dependent inhibition of CD16 expression (Fig 3B). This inhibitory effect is reversed by the addition of SPM (Fig 5B), suggesting that polyamine biosynthesis may be required for the conversion of CD14+CD16- monocytes into CD14+16+ monocytes. In addition, MGBG treatment decreased production of pro-inflammatory cytokines such as TNF-α produced by monocytes (S12 Fig). Thus, MGBG may attenuate the inflammatory response through regulation of monocyte differentiation. In this study we also found that MGBG interferes with monocyte DNA synthesis during differentiation. The BrdU incorporation experiment suggests that monocyte differentiation is accompanied by DNA synthesis and that MGBG interferes with this process. Although the role, if any, that DNA synthesis plays in monocyte differentiation remains to be clarified, the observation that these activities are inhibited by MGBG at similar doses and time of exposure suggests a linkage between these processes.

OPN is recognized as a Th1 pro-inflammatory cytokine and a major chemoattractant protein for monocyte migration [32, 33]. It has been reported that OPN is involved in macrophage survival and differentiation, and that OPN expression is required for maintenance of the macrophage differentiated phenotype [64]. OPN promotes the survival of activated and HIV-1 infected macrophages and thereby may promote the pathogenesis of HAD [65]. Therefore, MGBG mediated inhibition of OPN expression could interfere with the accumulation of macrophages generated from monocytes, as well as result in loss of pathogenic macrophages within diseased tissues. In HAD, this could lead to loss of HIV-1 infected macrophages, and perhaps reverse this aspect of the disease.

OPN has also been implicated in pathogenesis of obesity. It has been identified as a major player in monocyte chemotaxis, differentiation and local adipose tissue macrophage proliferation in obesity [66]. OPN promotes macrophage polarization towards M2 phenotype in experimental obesity [67]. The dose-dependent inhibitory effects of MGBG on OPN production, monocyte differentiation and BrdU uptake shown in this study imply a linkage among these activities. The effects of MGBG on monocyte differentiation and proliferation could be the result of a direct effect of the drug on OPN levels. MGBG also inhibits polyamine biosynthesis; however a specific role of polyamines, if any, in these processes remains to be defined. The effects of MGBG and OPN on monocyte/macrophage function deserve further study. The data presented here suggests that by interfering with OPN production, as well as monocyte differentiation and proliferation, MGBG might be useful for inhibiting macrophage driven processes in the pathogenesis of obesity.

The expression of OPN by mature macrophages is important in aspects of wound healing [28]; therefore it was essential to test whether the OPN regulatory effect of MGBG extended beyond the level of monocytes to mature macrophages. In experiments wherein monocyte cultures were exposed to MGBG at time points after initiation of culture, we found that the MGBG regulatory effect on OPN expression diminished after monocytes differentiated into mature macrophages (Fig 2B). Although MGBG efficiently enters both monocytes and macrophages [58], it functions more effectively at regulating OPN and CD16 expression in monocytes. This could be due to higher drug concentrations accumulating in the monocytes as compared to macrophages [58]. The relative monocyte-specific effects of MGBG allow the targeting of a narrow window of time in the monocyte differentiation process rather than mediating a global macrophage effect. The effect of MGBG on wound healing deserves extensive investigation.

The effect of MGBG on monocyte activation and polarization was further examined in studies where monocyte cultures were induced to become M1 or M2 macrophages compared with normal culture conditions. MGBG inhibited expression of differentiation markers of both M1 (CD64) and M2 macrophages (CD200R). However, expression of CD14, the pan monocyte/macrophage marker, was not significantly affected by the drug treatment. Therefore, MGBG appears to be a general inhibitor of monocyte differentiation and interfere with the initiation of macrophage polarization.

Inflammation plays an essential role in the pathogenesis of HIV-associated neurocognitive disorders (HAND). This is in part driven by HIV-infected macrophages with ongoing migration of activated monocytes into the central nervous system in the presence of elevated levels of OPN expression [68–70]. MGBG was tested for *in vivo* effects on disease activity in an animal model of AIDS dementia. In a simian immunodeficiency virus encephalopathy (SIVE) model wherein OPN expression in brain macrophages contributes to AIDS dementia pathogenesis, MGBG inhibits expression of OPN and SIV p28 expression within the brain, and appears moderately effective in preventing the macaques from developing SIVE [71]. MGBG also reduces monocyte activation, the dorsal root ganglia pathology, and inflammation in the SIVE model [72]. In this model, SIV infected macaques also developed myocarditis and early atherosclerosis, processes independent of the presence of infected cells within diseased tissues. MGBG inhibits this systemic manifestation of SIV infection [73]. Therefore, MGBG can apparently interfere with macrophage infection and secondary disease sequelae (SIVE) as well as interfere with disease pathogenesis associated with monocytic activation and migration contributing to inflammatory complications of SIV infection. These studies also demonstrated that MGBG given orally was non-toxic at the doses employed. The tissue levels of MGBG including those in the brain exceeded that required to cause the effects on monocyte differentiation demonstrated in the current study.

In this study we show that MGBG interferes with OPN expression in monocytes and inhibits monocyte differentiation into both M1 and M2 macrophages. The unique characteristics of MGBG, such as monocyte/macrophage specificity, the ability to cross blood-brain barrier, oral bioavailability, and without significant *in vivo* toxicity, make it an interesting candidate for drug development targeting diseases associated with enhanced macrophage activation and chronic inflammation.

## Supporting information

S1 Fig Gating strategy for FACS analysis. Freshly isolated or cultured human monocytes were stained with antibody or matching isotypes. For gating, cell debris was first excluded by forward and side scatters. In the FL1/3 or FL2/3 plots, CD14- cells were excluded based on the isotype staining. CD16 or iOPN geometric mean was obtained from the gated CD14+ cells. When cell percentage was used for calculation, cells were gated using quadrants.
(TIF)

S2 Fig FACS data for iOPN shown in Fig 1. (TIF)

S3 Fig FACS data for CD16 shown in Fig 3A. (TIF)

S4 Fig FACS data for CD16 shown in Fig 3B. (TIF)

S5 Fig FACS data for BrdU incorporation shown in Fig 3C. (TIF)

S6 Fig FACS data for 1D CD16 shown in Fig 4. (TIF)

S7 Fig FACS data for 3D CD16 shown in Fig 4. (TIF)

S8 Fig FACS data for 6D CD16 shown in Fig 4. (TIF)

S9 Fig FACS data for Fig 5B. (TIF)

S10 Fig FACS data for Fig 6. (TIF)

S11 Fig Monocytes are the main source of OPN in PBMC cultures. PBMCs, isolated CD14+ monocytes, and CD14- cells were cultured for 1, 3, 6 days for sOPN measurement. CD14+ monocytes/macrophages are the major OPN producing cells in non-stimulated PBMCs. n = 4, means and SEM.
(TIF)

S12 Fig MGBG decreased LPS-induced TNF-α in PBMC cultures. PBMCs, isolated CD14+ monocytes, and CD14- cells were cultured for 1 and 3 days with or without MGBG treatment. Cells were treated with 10 ng/ml LPS for 2 hours before culture supernatants being collected for TNF-α measurement. A. Monocytes are the major source of LPS-induced TNF-α cytokine in cultured PBMCs. n = 2. B. MGBG treatment decreased LPS-induced TNF-α in PBMC cultures. n = 2.
(TIF)

S1 Table P-values for Figs 1–5. (PDF)

## References

[1] Hume DA. The Many Alternative Faces of Macrophage Activation. Front Immunol. 2015 7 22;6:370 doi: 10.3389/fimmu.2015.00370 26257737 10.3389/fimmu.2015.00370 PMC4510422

[2] Wang N, Liang H, Zen K. Molecular mechanisms that influence the macrophage m1-m2 polarization balance. Front Immunol. 2014 11 28;5:614 doi: 10.3389/fimmu.2014.00614 25506346 10.3389/fimmu.2014.00614 PMC4246889

[3] Laria A, Lurati A, Marrazza M, Mazzocchi D, Re KA, Scarpellini M. The macrophages in rheumatic diseases. J Inflamm Res. 2016 2 9;9:1–11. doi: 10.2147/JIR.S82320 26929657 10.2147/JIR.S82320 PMC4755472

[4] Colin S, Chinetti-Gbaguidi G, Staels B. Macrophage phenotypes in atherosclerosis. Immunol Rev. 2014 11;262(1):153–66. doi: 10.1111/imr.12218 25319333 10.1111/imr.12218

[5] Schultze JL, Schmieder A, Goerdt S. Macrophage activation in human diseases. Semin Immunol. 2015 8;27(4):249–56. doi: 10.1016/j.smim.2015.07.003 26303100 10.1016/j.smim.2015.07.003

[6] Liu YC, Zou XB, Chai YF, Yao YM. Macrophage polarization in inflammatory diseases. Int J Biol Sci. 2014 5 1;10(5):520–9. doi: 10.7150/ijbs.8879 24910531 10.7150/ijbs.8879 PMC4046879

[7] Labonte AC, Tosello-Trampont AC, Hahn YS. The role of macrophage polarization in infectious and inflammatory diseases. Mol Cells. 2014 4;37(4):275–85. doi: 10.14348/molcells.2014.2374 24625576 10.14348/molcells.2014.2374 PMC4012075

[8] Passlick B, Flieger D, Ziegler-Heitbrock HWL. Identification and characterization of a novel monocyte subpopulation in human peripheral blood. Blood. 1989 11;74(7):2527–2534. 2478233

[9] Ziegler-Heitbrock HW, Fingerle G, Strobel M, Schraut W, Stelter F, Schutt C, et al The novel subset of CD14+/CD16+ blood monocytes exhibits features of tissue macrophages. Eur J Immunol. 1993 9;23(9):2053–2058. doi: 10.1002/eji.1830230902 7690321 10.1002/eji.1830230902

[10] Ziegler-Heitbrock HW. Heterogeneity of human blood monocytes: the CD14+ CD16+ subpopulation. Immunol Today. 1996 9;17(9):424–428. 8854561 10.1016/0167-5699(96)10029-3

[11] Gordon S, Taylor PR. Monocyte and macrophage heterogeneity. Nat Rev Immunol. 2005 12;5(12):953–964. doi: 10.1038/nri1733 16322748 10.1038/nri1733

[12] Zhou LJ, Tedder TF. CD14+ monocytes can differentiate into functionally mature CD83+ dendritic cells. Proc Natl Acad Sci USA. 1996 3;93(6):2588–2592. 8637918 10.1073/pnas.93.6.2588 PMC39841

[13] Brugger W, Kreutz M, Andreesen R. Macrophage colony-stimulating factor is required for human monocyte survival and acts as a cofactor for their terminal differentiation to macrophages in vitro. J Leukoc Biol. 1991 5;49(5):483–488. 2016569 10.1002/jlb.49.5.483

[14] Ancuta P, Rao R, Moses A, Mehle A, Shaw SK, Luscinskas FW, et al Fractalkine preferentially mediates arrest and migration CD16+ monocytes. J Exp Med. 2003 6;197(12):1701–1707. doi: 10.1084/jem.20022156 12810688 10.1084/jem.20022156 PMC2193954

[15] Clarkson SB, Ory PA. CD16. Developmentally regulated IgG Fc receptors on cultured human monocytes. J Exp Med. 1988 2;167(2):408–420. 2964496 10.1084/jem.167.2.408 PMC2188855

[16] Calzada-Wack JC, Frankenberger M, Ziegler-Heitbrock HW. Interleukin-10 drives human monocytes to CD16 positive macrophages. J Inflamm. 1996;46(2):78–85. 8734788

[17] Ziegler-Heitbrock L. The CD14+CD16+ blood monocytes: their role in infection and inflammation. J Leukoc Biol. 2007 3;81(3):584–592. doi: 10.1189/jlb.0806510 17135573 10.1189/jlb.0806510

[18] Belge KU, Dayyani F, Horelt A, Siedlar M, Frankenberger M, Frankenberger B, et al The proinflammatory CD14+CD16+DR++ monocytes are a major source of TNF. J Immunol. 2002 4;168(7):3536–3542. 11907116 10.4049/jimmunol.168.7.3536

[19] Kanai T, Makita S, Kawamura T, Nemoto Y, Kubota D, Naqayama K, et al Extracorporeal elimination of TNF-alpha-producing CD14(dull)CD16(+) monocytes in leukocytapheresis therapy for ulcerative colitis. Inflamm Bowel Dis. 2007 3;13(3):284–90. doi: 10.1002/ibd.20017 17206704 10.1002/ibd.20017

[20] Shinohara S, Hirohata S, Inoue T, Ito K. Phenotypic analysis of peripheral blood monocytes isolated from patients with rheumatoid arthritis. J Rheumatol. 1992 2;19(2):211–215. 1378495

[21] Zhang R, Miller RG, Madison C, Jin X, Honrada R, Harris W, et al Systemic immune system alterations in early stages of Alzheimer's disease. J Neuroimmunol. 2013 3 15;256(1–2):38–42. doi: 10.1016/j.jneuroim.2013.01.002 23380586 10.1016/j.jneuroim.2013.01.002 PMC3641776

[22] Saleh MN, Goldman SJ, LoBuglio AF, Beall AC, Sabio H, McCord MC, et al CD16+ monocytes in patients with cancer: spontaneous elevation and pharmacologic induction by recombinant human macrophage colony-stimulating factor. Blood. 1995 5;85(10):2910–2917. 7742551

[23] Pulliam L, Gascon R, Stubblebine M, McGuire D, McGrath MS. Unique monocyte subset in patients with AIDS dementia. Lancet. 1997 3;349(9053):692–695. doi: 10.1016/S0140-6736(96)10178-1 9078201 10.1016/S0140-6736(96)10178-1

[24] Tanaka M, Honda J, Imamura Y, Shiraishi K, Tanka K, Oizumi K. Surface phenotype analysis of CD16+ monocytes from leukapheresis collections for peripheral blood progenitors. Clin Exp Immunol. 1999 4;116(1):57–61. doi: 10.1046/j.1365-2249.1999.00869.x 10209505 10.1046/j.1365-2249.1999.00869.x PMC1905216

[25] Zhang R, Gascon R, Miller RG, Gelinas DF, Mass J, Hadlock K, et al Evidence for systemic immune system alterations in sporadic amyotrophic lateral sclerosis (sALS). J Neuroimmunol. 2005 2;159(1–2):215–224. doi: 10.1016/j.jneuroim.2004.10.009 15652422 10.1016/j.jneuroim.2004.10.009

[26] Shiramizu B, Gartner S, Williams A, Shikuma C, Ratto-Kim S, Watters M, et al Circulating proviral HIV DNA and HIV-associated dementia. AIDS. 2005 1;19(1):45–52. 15627032 10.1097/00002030-200501030-00005 PMC1557628

[27] Williams K, Westmoreland S, Greco J, Ratai E, Lentz M, Kim WK, et al Magnetic resonance spectroscopy reveals that activated monocytes contribute to neuronal injury in SIV neuroAIDS. J Clin Invest. 2005 9;115(9):2534–2545. doi: 10.1172/JCI22953 16110325 10.1172/JCI22953 PMC1187930

[28] Denhardt DT, Noda M, O’Regan AW, Pavlin D, Berman JS. Osteopontin as a means to cope with environmental insults: regulation of inflammation, tissue remodeling, and cell survival. J Clin Invest. 2001 5; 107(9):1055–1061. doi: 10.1172/JCI12980 11342566 10.1172/JCI12980 PMC209291

[29] Giachelli CM, Steutz S. Osteopontin: a versatile regulator of inflammation and biomineralizaton. Matrix Biol. 2000 12;19(7): 615–22. 11102750 10.1016/s0945-053x(00)00108-6

[30] Mazzali M, Kipari T, Ophascharoensuk V, Wesson JA, Johnson R, Hughes J. Osteopontin—a molecule for all seasons. QJM. 2002 1;95(1):3–13. 11834767 10.1093/qjmed/95.1.3

[31] Standal T, Borset M, Sundan A. Role of osteopontin in adhesion, migration, cell survival, and bone remodeling. Exp Oncol. 2004 9;26(3):179–184. 15494684

[32] Kahles F, Findeisen HM, Bruemmer D. Osteopontin: A novel regulator at the cross roads of inflammation, obesity and diabetes. Mol Metab. 2014 3 22;3(4):384–93. doi: 10.1016/j.molmet.2014.03.004 24944898 10.1016/j.molmet.2014.03.004 PMC4060362

[33] Brown A. Osteopontin: a key link between immunity, inflammation and the central nervous system. Transl Neurosci. 2012 2 1;3(3):288–293. doi: 10.2478/s13380-012-0028-7 23565338 10.2478/s13380-012-0028-7 PMC3616337

[34] Gimba ER, Tilli TM. Human osteopontin splicing isoforms: known roles, potential clinical applications and activated signaling pathways. Cancer Lett. 2013 4 30;331(1):11–7. doi: 10.1016/j.canlet.2012.12.003 23246372 10.1016/j.canlet.2012.12.003

[35] Inoue M, Shinohara ML. Intracellular osteopontin (iOPN) and immunity. Immunol Res. 2011 4;49(1–3):160–72. doi: 10.1007/s12026-010-8179-5 21136203 10.1007/s12026-010-8179-5 PMC3509172

[36] Rittling SR, Chamber AF. Role of osteopontin in tumor progression. Br J Cancer. 2004 5; 17;90(10):1877–81. doi: 10.1038/sj.bjc.6601839 15138464 10.1038/sj.bjc.6601839 PMC2410284

[37] Chakraborty G, Jain S, Behera R, Ahmed M, Sharma P, Kumar V, et al The multifaceted roles of osteopontin in cell signaling, tumor progression and angiogenesis. Curr Mol Med. 2006 12;6(8):819–30. 17168734 10.2174/156652406779010803

[38] Burdo TH, Ellis RJ, Fox HS. Osteopontin is increased in HIV-associated dementia. J Infect Dis. 2008 9;198(5):715–22. doi: 10.1086/590504 18616394 10.1086/590504 PMC2587027

[39] Brown A, Islam T, Adams R, Nerle S, Kamara M, Eger C, et al Osteopontin enhances HIV replication and is increased in the brain and cerebrospinal fluid of HIV-infected individuals. J Neurovirol. 2011 8;17(4):382–92. doi: 10.1007/s13365-011-0035-4 21556958 10.1007/s13365-011-0035-4 PMC3331788

[40] Chabas D, Baranzini SE, Mitchell D, Bernard CC, Rittling SR, Denhardt DT, et al The influence of the proinflammatory cytokine, osteopontin, on autoimmune demyelinating disease. Science. 2001 11 23; 294(5547):1731–5. doi: 10.1126/science.1062960 11721059 10.1126/science.1062960

[41] Hur EM, Youssef S, Haws ME, Zhang SY, Sobel RA, Steinman L. Osteopontin-induced relapse and progression of autoimmune brain disease through enhanced survival of activated T cells. Nat Immunol. 2007 1;8(1):74–83. doi: 10.1038/ni1415 17143274 10.1038/ni1415

[42] Petrow PK, Hummel KM, Schedel J, Franz JK, Klein CL, Muller-Ladner U, et al Expression of osteopontin messenger RNA and protein in rheumatoid arthritis: effects of osteopontin on the release of collagenase 1 from articular chondrocytes and synovial fibroblasts. Arthritis Rheum. 2000 7;43(7):1597–605. doi: 10.1002/1529-0131(200007)43:7<1597::AID-ANR25>3.0.CO;2-0 10902765 10.1002/1529-0131(200007)43:7<1597::AID-ANR25>3.0.CO;2-0

[43] Castoldi A, Naffah de Souza C, Câmara NO, Moraes-Vieira PM. The Macrophage Switch in Obesity Development. Front Immunol. 2016 1 5;6:637 doi: 10.3389/fimmu.2015.00637 26779183 10.3389/fimmu.2015.00637 PMC4700258

[44] Barchetta I, Alessandri C, Bertoccini L, Cimini FA, Taverniti L, Di Franco M, et al Increased circulating osteopontin levels in adult patients with type 1 diabetes mellitus and association with dysmetabolic profile. Eur J Endocrinol. 2016 2;174(2):187–92. doi: 10.1530/EJE-15-0791 26578639 10.1530/EJE-15-0791

[45] Renkl AC, Wussler J, Ahrens T, Thoma K, Kon S, Uede T, et al Osteopontin functionally activates dendritic cells and induces their differentiation toward a Th1-polarizing phenotype. Blood. 2005 8 1;106(3):946–55. doi: 10.1182/blood-2004-08-3228 15855273 10.1182/blood-2004-08-3228

[46] Jain S, Chakraborty G, Bulbule A, Kaur R, Kundu GC. Osteopontin: an emerging therapeutic target for anticancer therapy. Expert Opin Ther Targets. 2007 1;11(1):81–90. doi: 10.1517/14728222.11.1.81 17150036 10.1517/14728222.11.1.81

[47] Saito Y, Kon S, Fujiwara Y, Nakayama Y, Kurotaki D, Fukuda N, et al Osteopontin small interfering RNA protects mice from fulminant hepatitis. Hum Gene Ther. 2007 12;18(12):1205–14. doi: 10.1089/hum.2007.069 17988193 10.1089/hum.2007.069

[48] Dai J, Li B, Shi J, Peng L, Zhang D, Qian W, et al A humanized anti-osteopontin antibody inhibits breast cancer growth and metastasis in vivo. Cancer Immunol Immunother. 2010 3;59(3):355–66. doi: 10.1007/s00262-009-0754-z 19690854 10.1007/s00262-009-0754-z PMC11030624

[49] Kiefer FW, Zeyda M, Gollinger K, Pfau B, Neuhofer A, Weichhart T, et al Neutralization of osteopontin inhibits obesity-induced inflammation and insulin resistance. Diabetes. 2010 4;59(4):935–46. doi: 10.2337/db09-0404 20107108 10.2337/db09-0404 PMC2844841

[50] Boumans MJ, Houbiers JG, Verschueren P, Ishikura H, Westhovens R, Brouwer E, et al Safety, tolerability, pharmacokinetics, pharmacodynamics and efficacy of the monoclonal antibody ASK8007 blocking osteopontin in patients with rheumatoid arthritis: a randomised, placebo controlled, proof-of-concept study. Ann Rheum Dis. 2012 2;71(2):180–5. doi: 10.1136/annrheumdis-2011-200298 21917822 10.1136/annrheumdis-2011-200298

[51] Pegg AE, McCann PP. Polyamine metabolism and function. Am J Physiol. 1982 11;243(5):C212–21. doi: 10.1152/ajpcell.1982.243.5.C212 6814260 10.1152/ajpcell.1982.243.5.C212

[52] Jänne J, Hölttä E, Kallio A, Käpyaho K. Role of polyamines and their antimetabolites in clinical medicine. Spec Top Endocrinol Metab. 1983;5:227–93. 6367119

[53] Messina L, Spampinato G, Arcidiacono A, Malaquarnera L, Paqano M, Kaminska B, et al Polyamine involvement in functional activation of human macrophages. J Leukoc Biol. 1992 12;52(6):585–587. 1334500 10.1002/jlb.52.6.585

[54] Kaczmarek L, Kaminska B, Messina L, Spampinato G, Arcidiacono A, et al Inhibitors of polyamine biosynthesis block tumor necrosis factor-induced activation of macrophages. Cancer Res. 1992 4;52(7):1891–1894. 1312903

[55] Williams-Ashman HG, Schenone A. Methyl glyoxal bis(guanylhydrazone) as a potent inhibitor of mammalian and yeast S-adenosylmethionine decarboxylases. Biochem Biophys Res Commun. 1972 1 14;46(1):288–95. 4550082 10.1016/0006-291x(72)90661-4

[56] Corti A, Dave C, Williams-Ashman HG, Mihich E, Schenone A. Specific inhibition of the enzymic decarboxylation of S-adenosylmethionine by methylglyoxal bis(guanylhydrazone) and related substances. Biochem J. 1974 5;139(2):351–7. 4447616 10.1042/bj1390351 PMC1166290

[57] Pegg AE. Inhibition of spermidine formation in rat liver and kidney by methylglyoxal bis(guanylhydrazone). Biochem J. 1973 3;132(3):537–40. 4724588 10.1042/bj1320537 PMC1177618

[58] Jin X, McGrath MS, Xu H. Inhibition of HIV expression and integration in macrophages by methylglyoxal-bis-guanylhydrazone. J Virol. 2015 11;89(22):11176–89. doi: 10.1128/JVI.01692-15 26223636 10.1128/JVI.01692-15 PMC4645666

[59] Hadlock KG, Lancero H, Yu S, Do HK. Regulation of osteopontin. 2013 May.United states patent: US 8,445,540 B2.

[60] Wai PY, Kuo PC. The role of Osteopontin in tumor metastasis. J Surg Res. 2004 10;121(2):228–41. doi: 10.1016/j.jss.2004.03.028 15501463 10.1016/j.jss.2004.03.028

[61] Zheng W, Li R, Pan H, He D, Xu R, Guo TB, et al Role of osteopontin in induction of monocyte chemoattractant protein 1 and macrophage inflammatory protein 1beta through the NF-kappaB and MAPK pathways in rheumatoid arthritis. Arthritis Rheum. 2009 7;60(7):1957–65. doi: 10.1002/art.24625 19565503 10.1002/art.24625

[62] Lund SA, Wilson CL, Raines EW, Tang J, Giachelli CM, Scatena M. Osteopontin mediates macrophage chemotaxis via α4 and α9 integrins and survival via the α4 integrin. J Cell Biochem. 2013 5;114(5):1194–202. doi: 10.1002/jcb.24462 23192608 10.1002/jcb.24462 PMC12462639

[63] Santos-Alvarez J, Goberna R, Sánchez-Margalet V. Human leptin stimulates proliferation and activation of human circulating monocytes. Cell Immunol. 1999 5 25;194(1):6–11. doi: 10.1006/cimm.1999.1490 10357875 10.1006/cimm.1999.1490

[64] Nystrom T, Duner P, Hultgardh-Hilsson A. A constitutive endogenous osteopontin production is important for macrophage function and differentiation. Exp Cell Res. 2007 4 1;313(6):1149–60. doi: 10.1016/j.yexcr.2006.12.026 17306792 10.1016/j.yexcr.2006.12.026

[65] Burdo TH, Wood MR, Fox HS. Osteopontin prevents monocyte recirculation and apoptosis. J Leukoc Biol. 2007 6;81(6):1504–11. doi: 10.1189/jlb.1106711 17369493 10.1189/jlb.1106711 PMC2490714

[66] Tardelli M, Zeyda K, Moreno-Viedma V, Wanko B, Grün NG, Staffler G, et al Osteopontin is a key player for local adipose tissue macrophage proliferation in obesity. Mol Metab. 2016 9 13;5(11):1131–1137. doi: 10.1016/j.molmet.2016.09.003 27818939 10.1016/j.molmet.2016.09.003 PMC5081407

[67] Schuch K, Wanko B, Ambroz K, Castelo-Rosa A, Moreno-Viedma V, Grün NG, et al Osteopontin affects macrophage polarization promoting endocytic but not inflammatory properties. Obesity (Silver Spring). 2016 7;24(7):1489–98. 27221527 10.1002/oby.21510

[68] Hong S, Banks WA. Role of the immune system in HIV-associated neuroinflammation and neurocognitive implications. Brain Behav Immun. 2015 3;45:1–12. doi: 10.1016/j.bbi.2014.10.008 25449672 10.1016/j.bbi.2014.10.008 PMC4342286

[69] Brown A. Understanding the MIND phenotype: macrophage/microglia inflammation in neurocognitive disorders related to human immunodeficiency virus infection. Clin Transl Med. 2015 2 26;4:7 doi: 10.1186/s40169-015-0049-2 25852823 10.1186/s40169-015-0049-2 PMC4385031

[70] Burdo TH, Lackner A, Williams KC. Monocyte/macrophages and their role in HIV neuropathogenesis. Immunol Rev. 2013 7;254(1):102–13. doi: 10.1111/imr.12068 23772617 10.1111/imr.12068 PMC3704190

[71] Burdo TH, Walker J, Xu H, Miller AD, McGrath MS, Williams KC. SIV-Associated Pathogenesis Modulation with Macrophage Targeted MGBG. 2016 Conference on Retroviruses and Opportunistic Infections. CROI, Boston, MA.

[72] Lakritz JR, Yalamanchili S, Polydefkis MJ, Miller AD, McGrath MS, Williams KC, et al An oral form of methylglyoxal-bis-guanylhydrazone reduces monocyte activation and traffic to the dorsal root ganglia in a primate model of HIV-peripheral neuropathy. J Neurovirol. 2017 8;23(4):568–576. doi: 10.1007/s13365-017-0529-9 28462488 10.1007/s13365-017-0529-9 PMC5623097

[73] Walker JA, Miller AD, Burdo TH, McGrath MS, Williams KC. Direct Targeting of Macrophages With Methylglyoxal-Bis-Guanylhydrazone Decreases SIV-Associated Cardiovascular Inflammation and Pathology. J Acquir Immune Defic Syndr. 2017 4 15;74(5):583–592. doi: 10.1097/QAI.0000000000001297 28141779 10.1097/QAI.0000000000001297 PMC5370195

