# Supplementary figures and images for: Methylglyoxal-bis-guanylhydrazone inhibits osteopontin expression and differentiation in cultured human monocytes
Source: PLoS One. 2018 Mar 14;13(3):e0192680. doi: 10.1371/journal.pone.0192680 (PMC5851547; doi:10.1371/journal.pone.0192680)

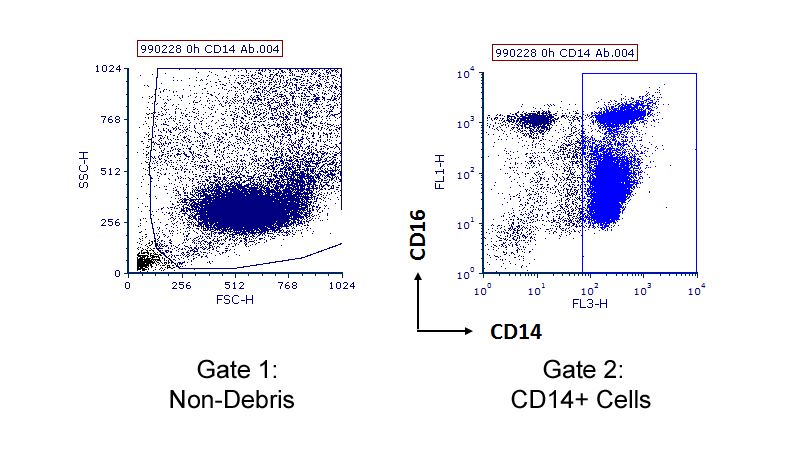

Supplement: S1 Fig — Freshly isolated or cultured human monocytes were stained with antibody or matching isotypes. For gating, cell debris was first excluded by forward and side scatters. In the FL1/3 or FL2/3 plots, CD14- cells were excluded based on the isotype staining. CD16 or iOPN geometric mean was obtained from the gated CD14+ cells. When cell percentage was used for calculation, cells were gated using quadrants. (TIF) [file pone.0192680.s001.tif]

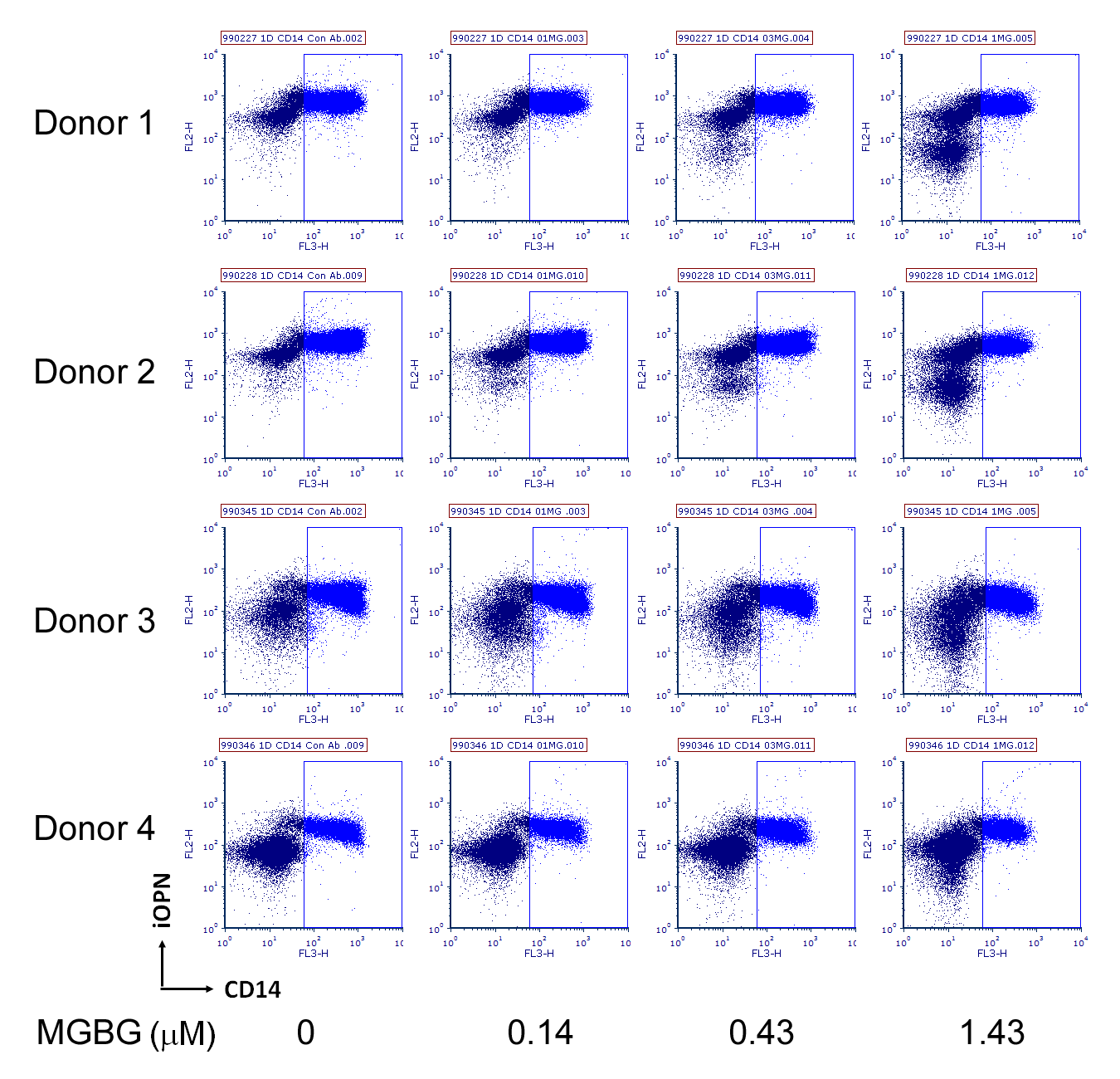

Supplement: S2 Fig — (TIF) [file pone.0192680.s002.tif]

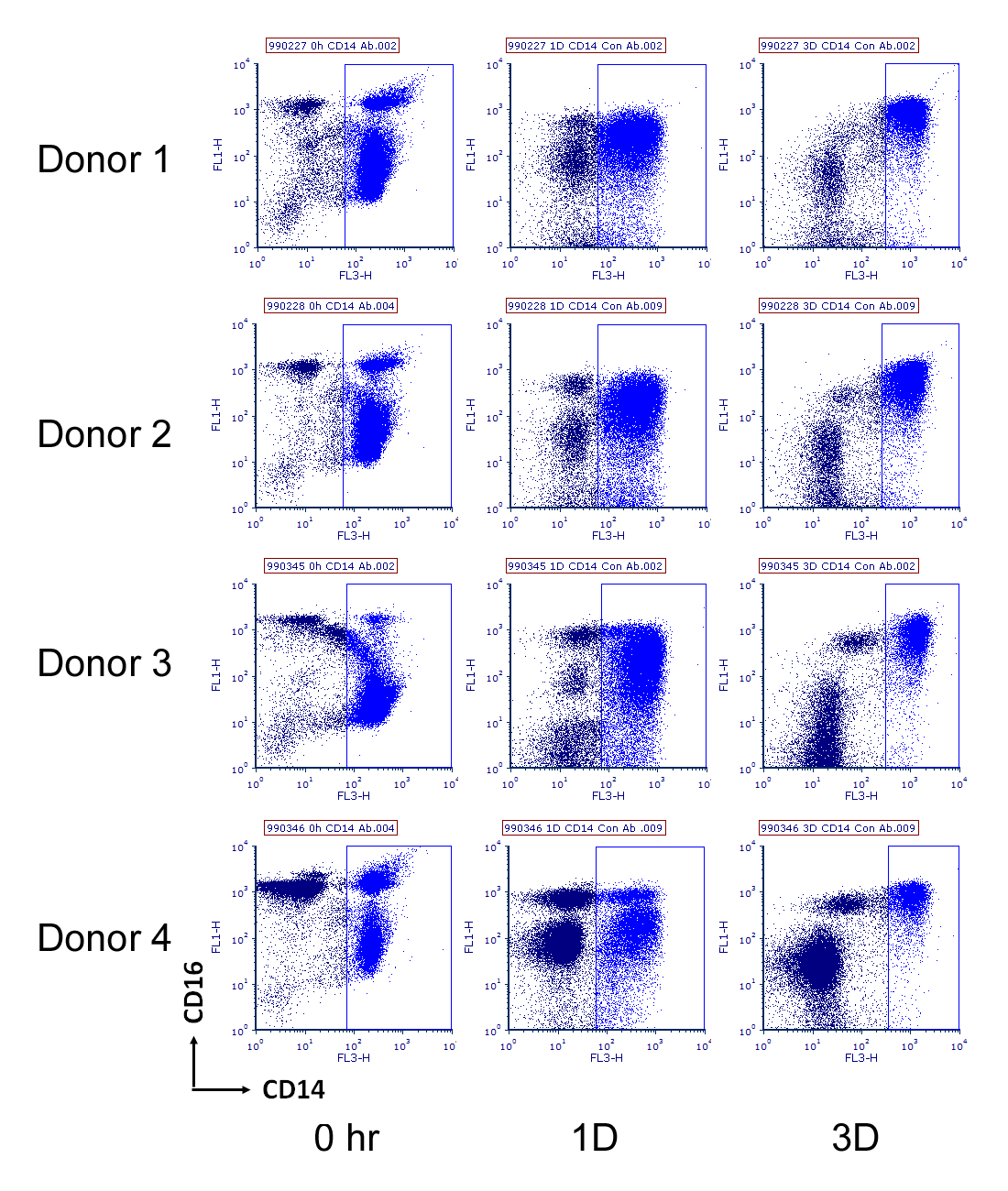

Supplement: S3 Fig — (TIF) [file pone.0192680.s003.tif]

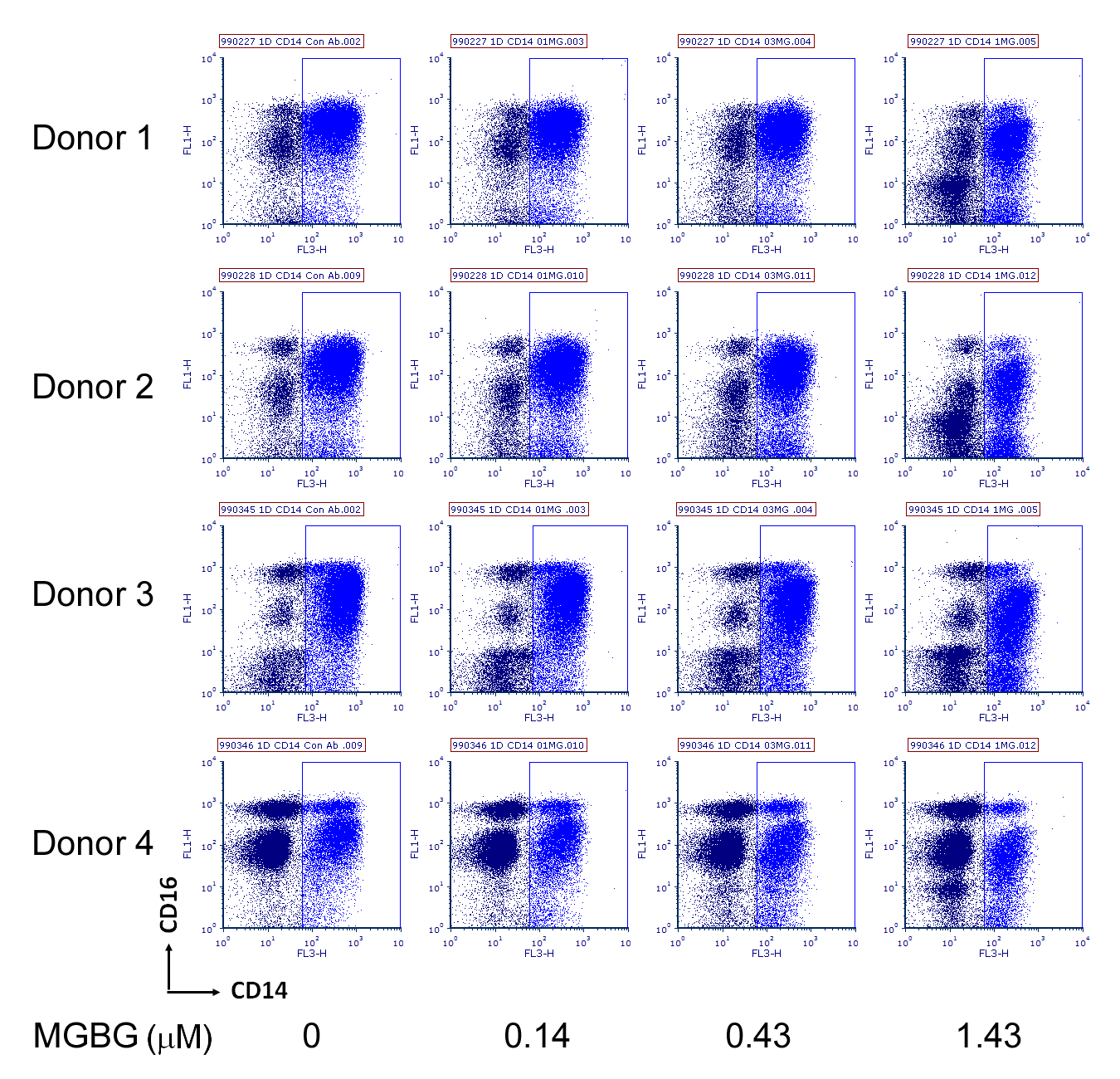

Supplement: S4 Fig — (TIF) [file pone.0192680.s004.tif]

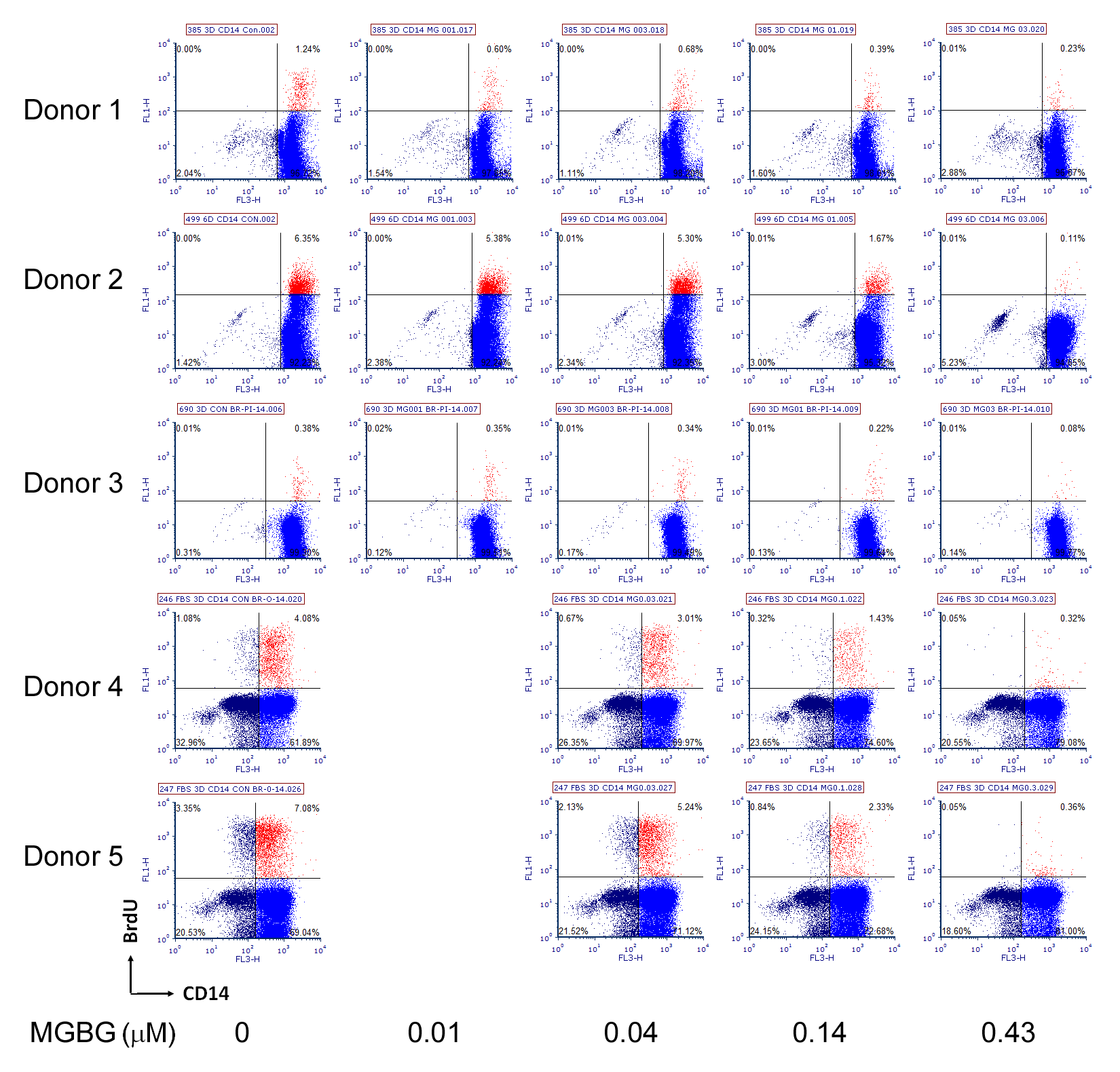

Supplement: S5 Fig — (TIF) [file pone.0192680.s005.tif]

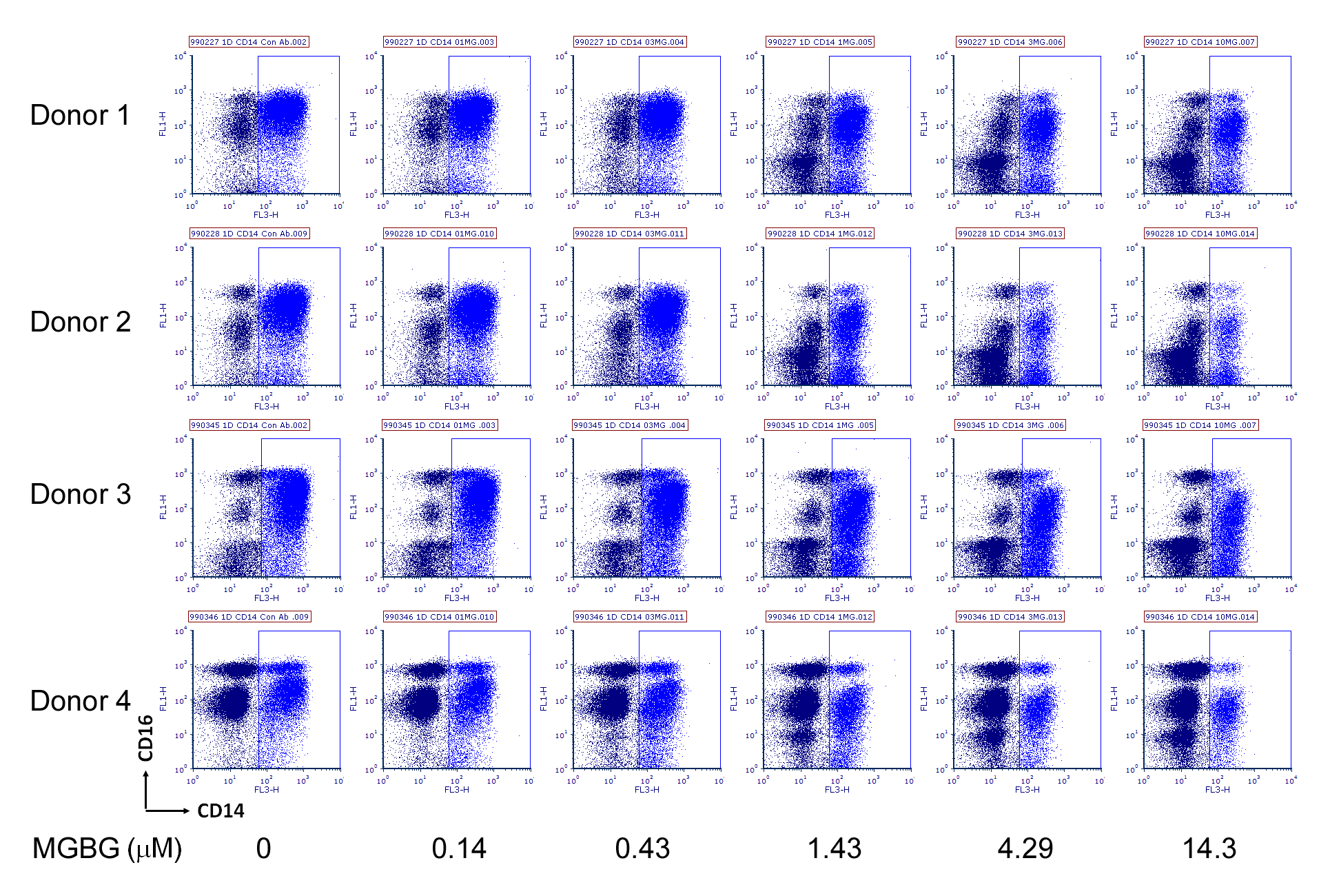

Supplement: S6 Fig — (TIF) [file pone.0192680.s006.tif]

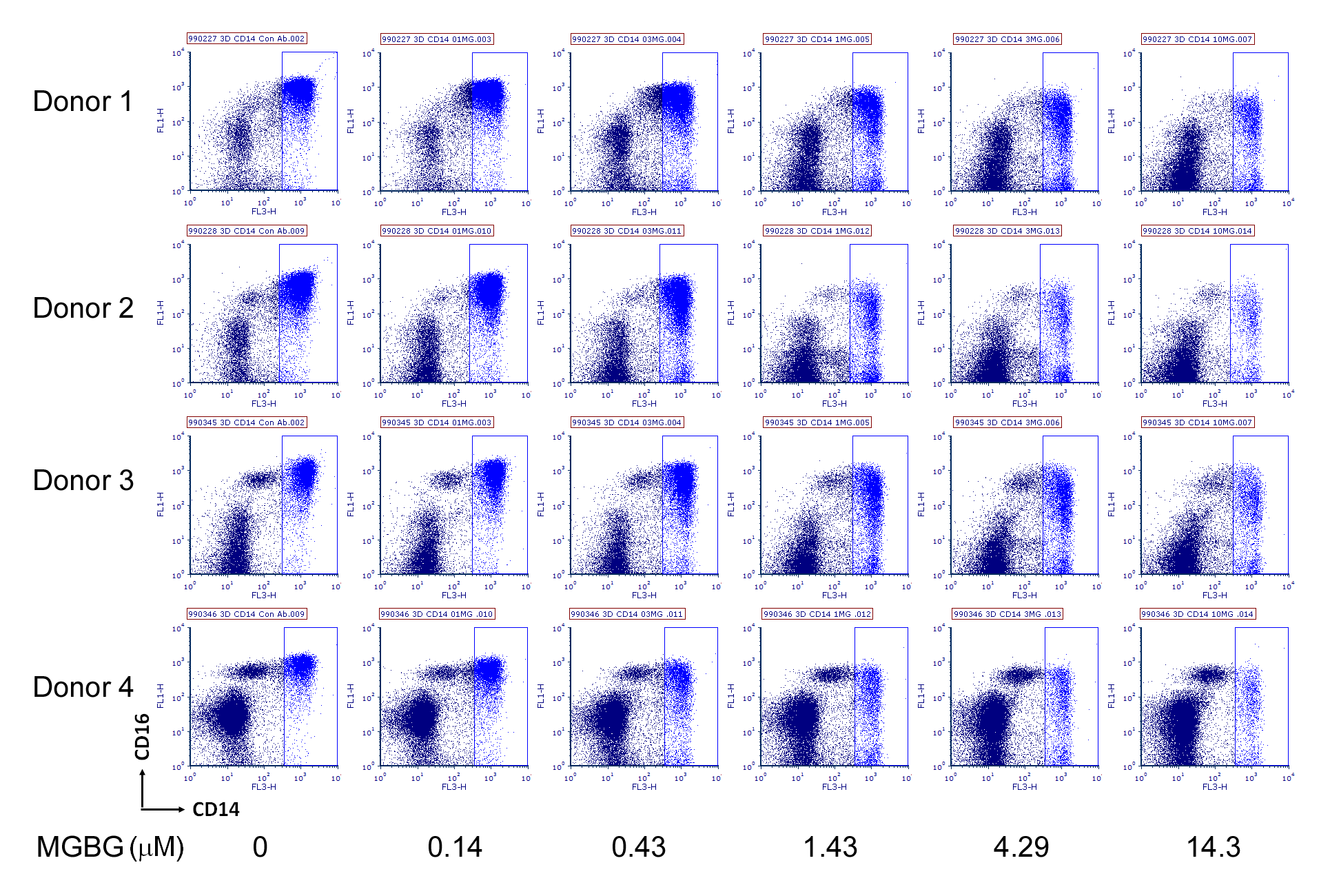

Supplement: S7 Fig — (TIF) [file pone.0192680.s007.tif]

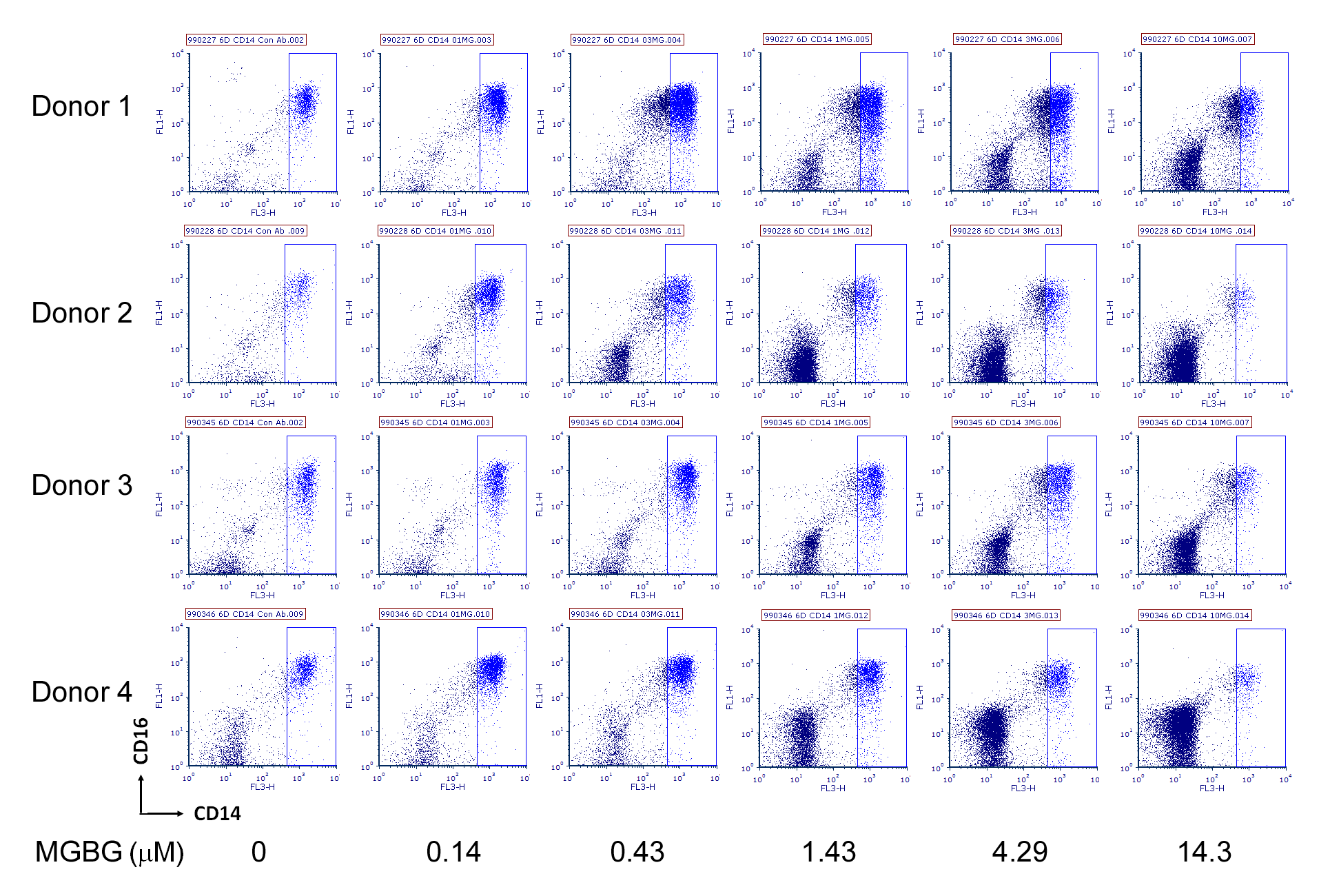

Supplement: S8 Fig — (TIF) [file pone.0192680.s008.tif]

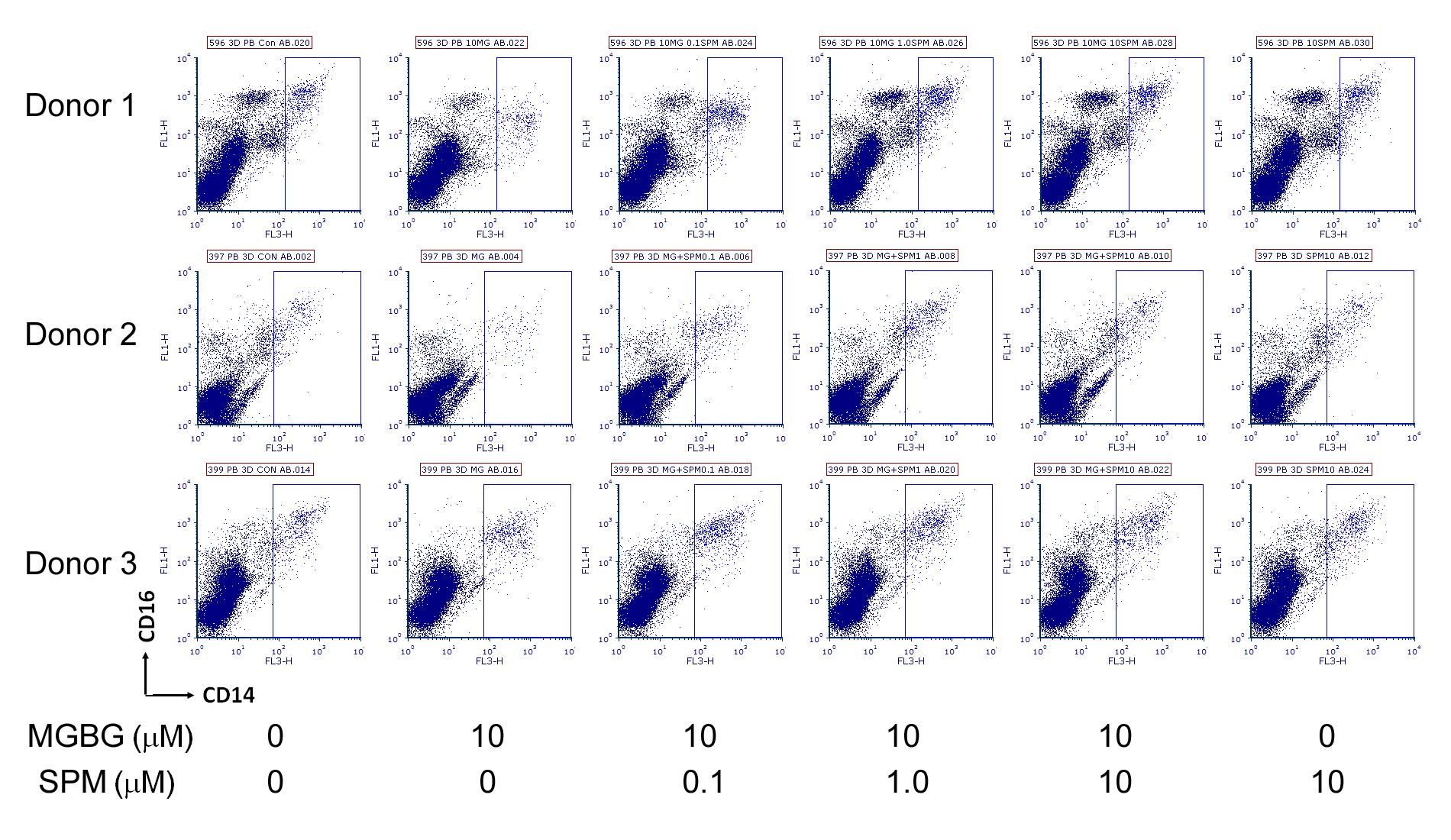

Supplement: S9 Fig — (TIF) [file pone.0192680.s009.tif]

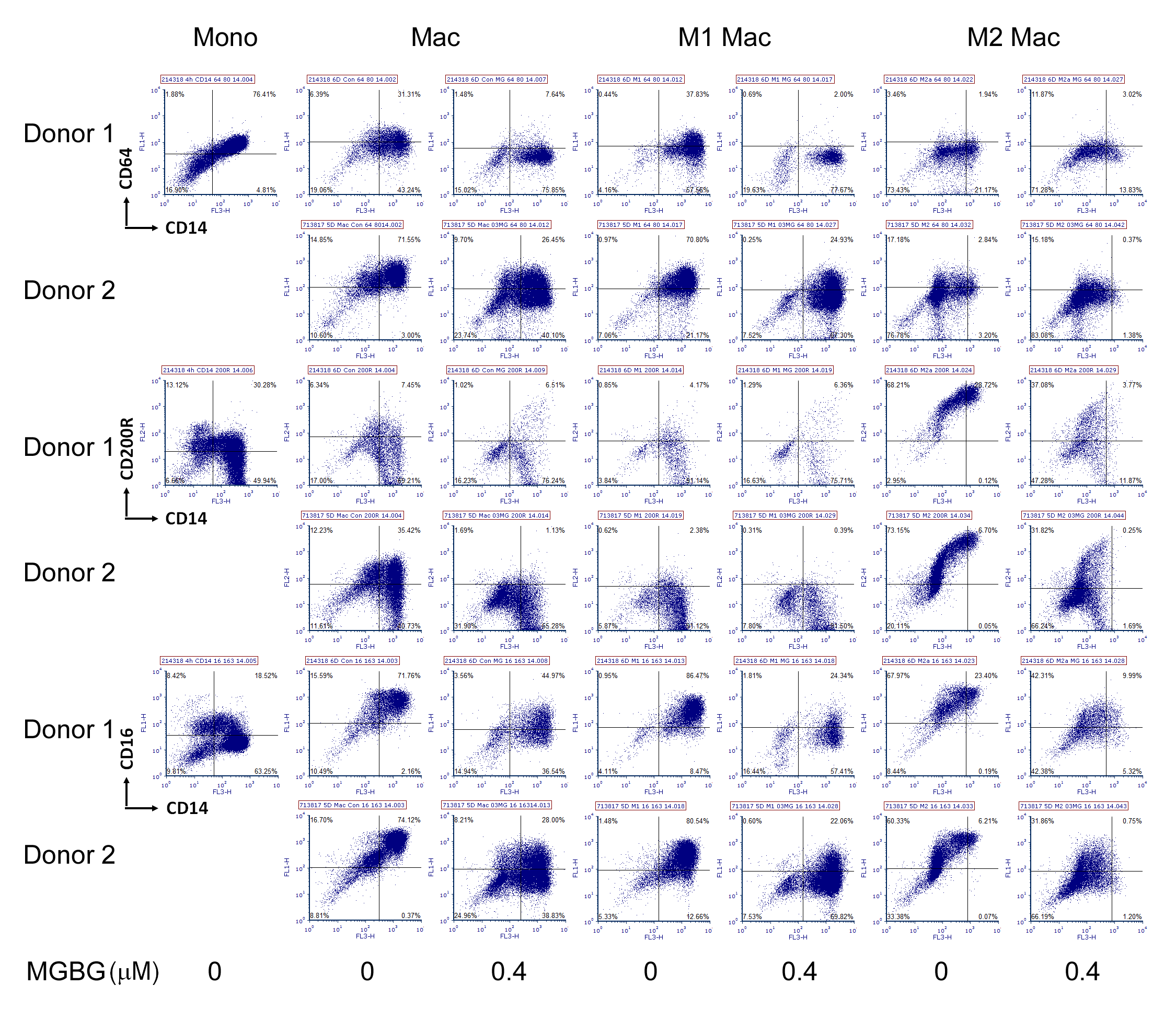

Supplement: S10 Fig — (TIF) [file pone.0192680.s010.tif]
