# Supplementary material for: Methylglyoxal-bis-guanylhydrazone inhibits osteopontin expression and differentiation in cultured human monocytes
Source: PLoS One. 2018 Mar 14;13(3):e0192680. doi: 10.1371/journal.pone.0192680 (PMC5851547; doi:10.1371/journal.pone.0192680)
Supplement: S1 Table — (PDF) [file pone.0192680.s013.pdf]

	P-Value
**Figure 1**	
sOPN 0 vs 0.14 µM MGBG	0.0037
sOPN 0 vs 0.43 µM MGBG	< 0.0001
sOPN 0 vs 1.42 µM MGBG	0.0001
iOPN 0 vs 0.14 µM MGBG	0.3147
iOPN 0 vs 0.43 µM MGBG	0.0129
iOPN 0 vs 1.42 µM MGBG	0.0574
OPN RNA 0 vs 0.14 µM MGBG	< 0.0001
OPN RNA 0 vs 0.43 µM MGBG	< 0.0001
OPN RNA 0 vs 1.42 µM MGBG	0.0024
**Figure 2A**	N/A
**Figure 2B**	
3D Mac 0 vs 10 µM MGBG	0.0819
**Figure 3A**	
CD 16 0 vs 1D	0.3453
CD16 0 vs 3D	0.0197
**Figure 3B**	
CD16 0 vs 0.14 µM MGBG	0.0042
CD16 0 vs 0.43 µM MGBG	0.0007
CD16 0 vs 1.42 µM MGBG	0.0002
**Figure 3C**	
BrdU 0 vs 0.01 µM MGBG	0.1816
BrdU 0 vs 0.04 µM MGBG	0.0138
BrdU 0 vs 0.14 µM MGBG	0.0003
BrdU 0 vs 0.43 µM MGBG	< 0.0001

	P-Value
**Figure 4**	
1D CD16 0 vs 0.14 µM MGBG	0.0042
1D CD16 0 vs 0.43 µM MGBG	0.0007
1D CD16 0 vs 1.42 µM MGBG	0.0002
1D CD16 0 vs 4.29 µM MGBG	0.0007
1D CD16 0 vs 14.3 µM MGBG	0.0005
3D CD16 0 vs 0.14 µM MGBG	0.1069
3D CD16 0 vs 0.43 µM MGBG	0.0013
3D CD16 0 vs 1.42 µM MGBG	< 0.0001
3D CD16 0 vs 4.29 µM MGBG	< 0.0001
3D CD16 0 vs 14.3 µM MGBG	< 0.0001
6D CD16 0 vs 0.14 µM MGBG	0.8587
6D CD16 0 vs 0.43 µM MGBG	0.3859
6D CD16 0 vs 1.42 µM MGBG	0.1866
6D CD16 0 vs 4.29 µM MGBG	0.1276
6D CD16 0 vs 14.3 µM MGBG	0.0459
**Figure 5A**	
OPN 0 vs 0.1 µM SPM	0.0060
OPN 0 vs 1.0 µM SPM	< 0.0001
OPN 0 vs 10 µM SPM	< 0.0001
**Figure 5B**	
CD16 0 vs 0.1 µM SPM	0.0738
CD16 0 vs 1.0 µM SPM	0.0016
CD16 0 vs 10 µM SPM	< 0.0001
**Figure 6**	N/A

P < 0.05: Significant
